# CMTM6 expressed on the adaxonal Schwann cell surface restricts axonal diameters in peripheral nerves

**DOI:** 10.1038/s41467-020-18172-7

**Published:** 2020-09-09

**Authors:** Maria A. Eichel, Vasiliki-Ilya Gargareta, Elisa D’Este, Robert Fledrich, Theresa Kungl, Tobias J. Buscham, Katja A. Lüders, Cristina Miracle, Ramona B. Jung, Ute Distler, Kathrin Kusch, Wiebke Möbius, Swen Hülsmann, Stefan Tenzer, Klaus-Armin Nave, Hauke B. Werner

**Affiliations:** 1 Department of Neurogenetics, Max Planck Institute of Experimental Medicine, 37075 Göttingen, Germany; 2 Department of Nanobiophotonics, Max Planck Institute for Biophysical Chemistry, 37077 Göttingen, Germany; 3 Optical Microscopy Facility, Max Planck Institute for Medical Research, 69120 Heidelberg, Germany; 4 Institute of Anatomy, University of Leipzig, 04103 Leipzig, Germany; 5 Institute of Immunology, University Medical Center, Johannes Gutenberg University, 55131 Mainz, Germany; 6 Focus Program Translational Neuroscience, University Medical Center, Johannes Gutenberg University, 55131 Mainz, Germany; 7 Electron Microscopy Core Unit, Max Planck Institute of Experimental Medicine, 37075 Göttingen, Germany; 8 Clinic for Anesthesiology, University Medical Center, 37073 Göttingen, Germany

**Keywords:** Cellular neuroscience, Schwann cell, Peripheral nervous system

## Abstract

The velocity of nerve conduction is moderately enhanced by larger axonal diameters and potently sped up by myelination of axons. Myelination thus allows rapid impulse propagation with reduced axonal diameters; however, no myelin-dependent mechanism has been reported that restricts radial growth of axons. By label-free proteomics, STED-microscopy and cryo-immuno electron-microscopy we here identify CMTM6 (chemokine-like factor-like MARVEL-transmembrane domain-containing family member-6) as a myelin protein specifically localized to the Schwann cell membrane exposed to the axon. We find that disruption of *Cmtm6*-expression in Schwann cells causes a substantial increase of axonal diameters but does not impair myelin biogenesis, radial sorting or integrity of axons. Increased axonal diameters correlate with accelerated sensory nerve conduction and sensory responses and perturbed motor performance. These data show that Schwann cells utilize CMTM6 to restrict the radial growth of axons, which optimizes nerve function.

## Abstract (web summary)

Myelinating cells differentially myelinate axons of different diameters, however whether they can also restrict radial axonal growth remained unclear. Here, the authors show that CMTM6 in Schwann cells restricts axon diameters, affecting sensory nerve conduction and behavioral performance.

## Introduction

By facilitating saltatory propagation of nerve impulses^1,2^ myelination of axons accelerates nerve conduction 20–100-fold^3^. In the peripheral nervous system (PNS), saltatory conduction requires the close cellular association between myelinated axons and myelinating Schwann cells^4,5^. Indeed, the development of functional axon/myelin-units involves complex signaling between neurons/axons, Schwann cells and the extracellular matrix^5,6^. A critical morphogenetic prerequisite for myelination is the radial sorting of axons out of bundles^7^. At this stage, axons that have a threshold diameter of 1 µm are sorted out by immature Schwann cells for subsequent individual myelination. It is thought that receptor tyrosine kinases (erbB2, erbB3) on Schwann cells sense and integrate the abundance of neuregulin-1 type-III on the axonal surface as a measure of its diameter^8^. Several additional signaling molecules steer peripheral myelination, including the G-protein-coupled receptor ADGRG6/GPR126, neurotrophic factors, integrin β1, and Schwann cell-derived LGI4 interacting with axonal ADAM22^9–13^. Thus, multiple extrinsic factors regulate the formation of myelin by Schwann cells. Less is known about signaling from Schwann cells to axons. For example, mice lacking myelin-associated glycoprotein (MAG) display reduced diameters^14^ and moderate degeneration^15^ of myelinated peripheral axons. MAG is an adaxonal Schwann cell protein, which is thought to signal towards the myelinated axon through its five immunoglobulin-like domains^14^.

Besides the strong effect of myelination^1,2^, the velocity of nerve conduction is also influenced by axonal diameters, internode length, and features of the nodes of Ranvier, though more moderately^16–19^. For example, the conduction speed along non-myelinated fibers increases roughly proportional to the square root of the axonal diameter^20,21^. To achieve a 10-fold increase in conduction speed, non-myelinated axons thus would have to be about 100 times larger^3^. In contrast, nerve conduction velocity (NCV) along myelinated fibers depends on linearly on axonal diameters^19,21,22^. Any variation of axonal diameters thus has a much more influential impact on NCV along myelinated compared to non-myelinated fibers. Theoretically, myelination allows rapid nerve conduction with reduced axonal diameters. However, by now no myelin-dependent mechanism has been reported to restrict the radial growth of axons. Here, we find that that the diameters of peripheral axons are indeed restricted by Schwann cells via a mechanism involving CMTM6, a transmembrane protein localized at their adaxonal surface.

## Results

### Proteome analysis of an axogliasome-enriched nerve fraction

**Fig. 1:**
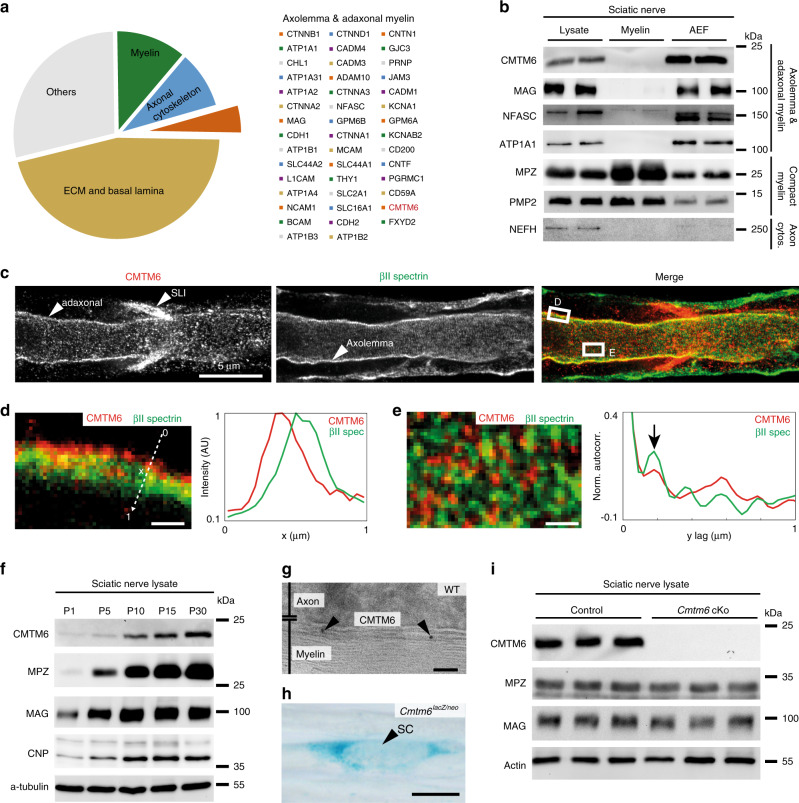
Identification of CMTM6 as adaxonal Schwann cell protein. **a** Quantitative proteome analysis of axogliasome-enriched fraction (AEF) from mouse sciatic nerves discovers CMTM6 (see Supplementary Fig. 1, 2 and Supplementary Data 1). **b** Immunoblot-analysis identifies enrichment of CMTM6 in the AEF compared to nerve lysate and myelin similar to markers for axolemma/adaxonal myelin (ATP1A1/NFASC/MAG). **c** STED-micrographs of teased fibers immunolabeled for CMTM6 (red) and betaII-spectrin (green). White arrowheads point at adaxonal myelin, Schmidt–Lanterman-incisure (SLI) and axolemma. Boxes indicate areas enlarged for line profile of intensities (**d**) and autocorrelation analysis (**e**). Scale bar, 5 µm. Age of mice 4–5 months. Cross section Supplementary Fig. 3a. **d** Line profile shows CMTM6-immunolabeling (red) adjacent to axolemma identified by betaII-spectrin-immunolabeling (green). Scale bar, 500 nm. **e** Autocorrelation analysis reveals a peak of periodicity of CMTM6 immunolabeling (red) longitudinally along the axon/myelin unit similar to betaII-spectrin-immunolabeling (green), indicated by black arrow. Scale bar, 500 nm. **f** Immunoblot-analysis shows developmentally increasing abundance of CMTM6 in sciatic nerves similar to myelin markers (MPZ, MAG, CNP). **g** Immunogold labeling of cross-sectioned sciatic nerve shows CMTM6-labeling (black particles; indicated by black arrowheads) confined to adaxonal myelin. Scale bar, 100 nm. **h** X-Gal-histochemistry on teased fibers shows activity of the *Cmtm6*^*lacZ/neo*^-allele (Supplementary Fig. 4a) in Schwann cells (indicated by black arrowhead). Scale bar, 25 µm. **i** Immunoblot-analysis reveals CMTM6-deficiency in sciatic nerves of *Cmtm6*-cKo mice (Supplementary Fig. 4). Blot shows *n* = 3 mice per genotype. Teased fiber labeling Supplementary Fig. 3b.

We hypothesized that the close association of peripheral axons and Schwann cells involves functionally important, but yet unknown proteins at the axon/myelin-interface. Aiming to discover novel proteins that mediate interactions between Schwann cells and axons, we biochemically purified a light-weight membrane fraction from sciatic nerves of wild-type mice (Supplementary Fig. 1). This method was originally established for the CNS, yielding a membrane fraction termed myelin–axolemmal complex^23^ or axogliasome^24^. Subjecting our fraction to quantitative mass spectrometry, we identified 755 proteins (Supplementary Data 1) including known markers of axolemma and adaxonal myelin membranes (Fig. 1a). Notably, extracellular matrix and basal lamina, compact myelin and axonal cytoskeleton were also present in the fraction (Fig. 1a and Supplementary Fig. 2), which we therefore renamed “axogliasome-enriched fraction” (AEF).

### Identification of CMTM6 as an adaxonal Schwann cell protein

Among the identified proteins, CMTM6 (chemokine-like factor-like MARVEL-transmembrane domain-containing family member-6) constitutes an average relative protein amount of 50 PPM (10 %CV) in the AEF according to quantitative proteome analysis (Supplementary Data 1). We selected CMTM6 for further analysis because immunoblot validated its enrichment in the AEF compared to nerve lysate (Fig. 1b) similar to markers of axolemma and adaxonal myelin (ATP1A1, MAG, NFASC). By STED microscopy on sciatic nerve teased fiber preparations, CMTM6 localizes to Schmidt–Lanterman-incisures (SLI) and adaxonal myelin (Fig. 1c, d and Supplementary Fig. 3a), in which it displays periodic distribution with an autocorrelation peak visible at 200 nm (Fig. 1e). Cryo-immuno electron microscopy confirmed adaxonal localization of CMTM6 (Fig. 1g). By immunoblot-analysis of sciatic nerves, CMTM6 displayed developmentally increasing abundance (Fig. 1f) similar to myelin markers (MPZ, MAG, CNP). When subjecting teased fiber preparations of *Cmtm6*^*lacZ/neo*^-mice (Supplementary Fig. 4a) to X-Gal-histochemistry (Fig. 1h), the labeling pattern indicated gene expression in Schwann cells. We then used *Dhh*^*Cre*^-driver-mice^25^ for recombination of an engineered *Cmtm6*^*flox*^-allele (Supplementary Fig. 4) to delete CMTM6-expression specifically in Schwann cells. Indeed, qRT-PCR (Supplementary Fig. 4c), immunoblotting (Fig. 1i) and immunolabeling of teased fiber preparations (Supplementary Fig. 3b) confirmed the absence of CMTM6-expression from sciatic nerves in *Cmtm6*^*flox/flox*^;*Dhh*^*Cre*^-mice (also termed conditional knockout, cKo, in the following). Together, these data indicate that CMTM6 is a Schwann cell protein localized to the adaxonal myelin membrane.

### Increased diameters of peripheral axons in *Cmtm6*-cKO mice

**Fig. 2:**
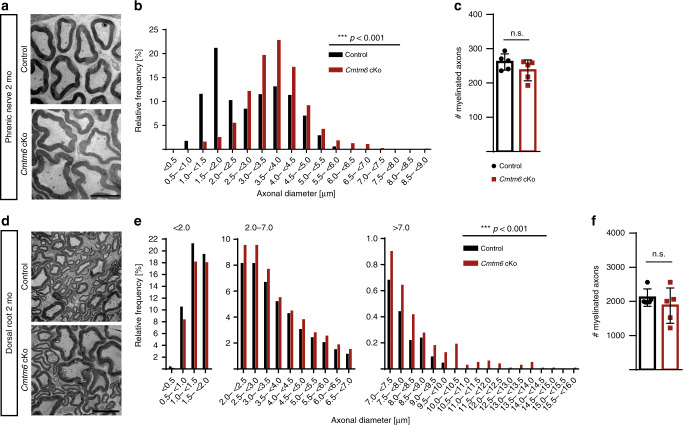
Diameters of myelinated axons are abnormally increased when CMTM6 is lacking from Schwann cells. **a** Electron micrographs of cross-sectioned phrenic nerves reveal increased axonal diameters in *Cmtm6-*cKo compared to control mice at 2 months (2 mo). Scale bar, 5 µm. **b** Genotype-dependent assessment of myelinated axons on semi-thin sections confirms shift towards larger axonal diameters in phrenic nerves of *Cmtm6*-cKO compared to control mice. Data are presented as frequency distribution with 0.5 µm bin width. *n* = 1293 axons from *n* = 5 control mice and *n* = 1172 axons from *n* = 5 *Cmtm6*-cKO mice; Mean axonal diameter (control+/−*Cmtm6-cKo*) = 2.87 µm + 0.84 µm; *P* = 2.2e^−16^ by two-sided Kolmogorow–Smirnow test of frequency distributions. For sciatic nerves and tail nerves, Supplementary Fig. 5**;** *g*-ratios Supplementary Fig. 6**;** nodal parameters Supplementary Fig. 7; neurofilament density Supplementary Fig. 8**;** tamoxifen-induced deletion in adult mice Fig. 4. **c** Genotype-dependent quantification on semi-thin sections shows unchanged number of myelinated axons in the phrenic nerves of *Cmtm6*-cKo-mice. *n* = 5 mice per genotype; *P* = 0.1929 by two-tailed Student’s *t*-test. **d** Representative electron micrographs of cross-sectioned dorsal roots show increased axonal diameters in *Cmtm6-*cKo compared to control mice at 2 mo. Scale bar 5 µm. **e** Genotype-dependent assessment of the diameters of myelinated axons on semi-thin sections confirms the shift towards larger axonal diameters in dorsal roots of *Cmtm6*-cKO-mice. Data are presented as frequency distribution with 0.5 µm bin width, *n* = 10387 axons from *n* = 5 control mice and *n* = 9298 axons from *n* = 5 *Cmtm6*-cKO mice; mean axonal diameter (control+/−*Cmtm6-cKo*) = 2.47 µm + 0.27 µm; P = 2.2e^−^^16^ by two-sided Kolmogorow–Smirnow test of frequency distributions. **f** Genotype-dependent quantification on semi-thin sections shows unchanged numbers of myelinated axons in the dorsal root of *Cmtm6*-cKo-mice. *n* = 5 mice per genotype; *P* = 0.3894 by two-tailed Student’s *t*-test. Data in **c** and **f** presented as mean ± SD. n.s. = non-significant *P* > 0.05; ***P < 0.001. Source data see Source Data file.

Hypothesizing that CMTM6 mediates important functional interactions between Schwann cells and axons, we assessed the consequences of its deletion in *Cmtm6*-cKo-mice. Strikingly, *Cmtm6*-cKo-mice displayed strongly increased axonal diameters in phrenic nerves (Fig. 2a, b), dorsal roots (Fig. 2d, e), sciatic nerves (Supplementary Fig. 5a, b) and ventral tail nerves (Supplementary Fig. 5f). Axonal loss was not a feature (Fig. 2c, f and Supplementary Fig. 5c, e, g), at least up to an age of 2 months. This indicates a role for CMTM6 in restricting the radial growth of axonal diameters. Importantly, the ratio between myelin sheath thickness and axonal diameter in *Cmtm6*-cKo-mice was appropriate as indicated by normal *g*-ratios in all assessed nerves and ages (Supplementary Fig. 6).

### Nerve conduction velocity and behavioral performance

**Fig. 3:**
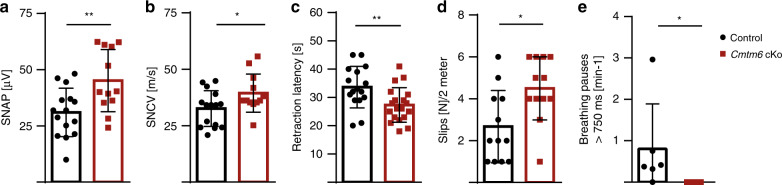
Electrophysiological properties and behavioral performance of mice lacking CMTM6 from Schwann cells. **a**, **b** Electrophysiological measurement reveals a larger sensory nerve action potential (SNAP) and accelerated sensory nerve conduction velocity (SNCV) in the tails of *Cmtm6*-cKo compared to control mice at P75. *n* = 15 control and *n* = 12 *Cmtm6-*cKo mice; **a** *P* = 0.0061 and **b** *P* = 0.0386 by two-tailed Student’s *t*-test. **c** The latency of retracting a hindlimb upon a heat stimulus was reduced in *Cmtm6*-cKo compared to control mice. *n* = 16 control and *n* = 19 *Cmtm6-*cKo mice; *P* = 0.0089 by two-tailed Student’s *t*-test. **d** Compared to control mice, *Cmtm6-*cKo-mice showed an increased number of fore- and hindlimb slips while traveling a distance of 2 m on a regular grid. *n* = 12 mice per genotype; *P* = 0.0111 by two-tailed Student’s *t*-test. **e** *Cmtm6-*cKo-mice did not show breathing pauses longer than 750 ms. *n* = 6 mice per genotype; *P* = 0.0152 by two-sided Mann–Whitney rank-sum test. Data presented as mean ± SD; *P < 0.05; **P < 0.01. Source data see Source Data file.

Considering that nerve conduction velocity (NCV) in myelinated fibers is roughly linear proportional to axonal diameter and myelin sheath thickness^3,17,18^, we assessed NCV in peripheral nerves of *Cmtm6*-cKo-mice. Indeed, sensory nerve action potentials (SNAP) were enlarged, and sensory nerve conduction velocity (SNCV) was accelerated in *Cmtm6*-cKo-mice (Fig. 3a, b). Internode length, nodal and paranodal dimensions may also affect NCV^16–19^ but were unaltered in *Cmtm6*-cKo-mice (Supplementary Fig. 7), implying that the increased NCV is owing to the increased axonal diameters and myelin thickness.

We then tested whether CMTM6-deficiency alters behavioral performance. Indeed, *Cmtm6*-cKo-mice displayed accelerated sensory reaction on the hot plate (Fig. 3c) and increased slipping frequency when walking over a regular grid (Fig. 3d). We also assessed breathing of *Cmtm6*-cKo-mice considering the increased axonal diameters in the phrenic nerve (Fig. 2a, b), which controls respiration. Wild-type C57BL/6N mice display breathing pauses of >750 ms/s termed spontaneous apnea^26^. Notably, these breathing pauses were absent in *Cmtm6-*cKo-mice (Fig. 3e). Together, increased axonal diameters are a plausible explanation for increased NCV and altered behavioral performance of *Cmtm6-*cKo-mice.

Radial axonal growth is frequently associated with altered density and phosphorylation of neurofilaments^27^. However, by electron microscopy (Supplementary Fig. 8a, b) and immunoblotting (Supplementary Fig. 8c–f), *Cmtm6*-cKo-mice displayed unaltered neurofilament density and phosphorylation, implying that the increase of axonal diameters reflects genuine radial growth.

### Increased axonal diameters in tamoxifen-inducible *Cmtm6*-cKo

**Fig. 4:**
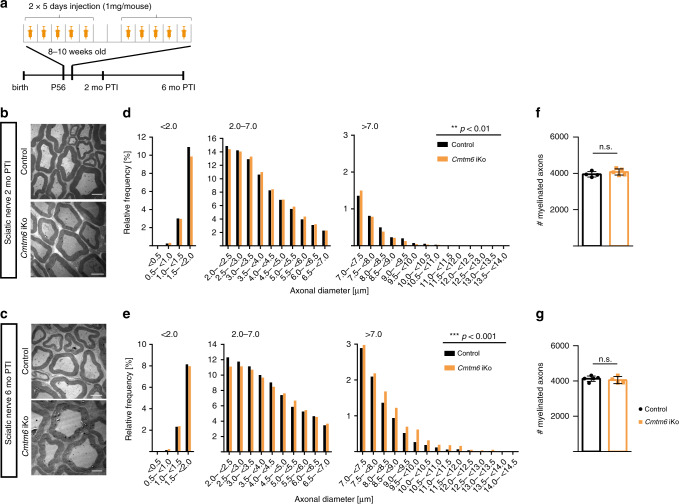
Adult deletion of *Cmtm6* causes increased axonal diameters. **a** Scheme for tamoxifen injection and time points post tamoxifen injection (PTI) for analyzing control mice (genotype *Cmtm6*^*flox/flox*^) and *Cmtm6*-iKo-mice (genotype *Cmtm6*^*flox/flox*^;*Plp*^*CreERT*2^). **b**, **c** Representative electron micrographs of cross-sectioned sciatic nerves show increased axonal diameters in *Cmtm6*-iKo compared to control mice 2 mo PTI (**b**) and 6 mo PTI (**c**). Scale bar, 2.5 µm. **d**, **e** Genotype-dependent quantification of diameters of myelinated axons in sciatic nerves dissected from *Cmtm6*-iKo and control mice 2 mo PTI (**d**) or 6 mo PTI (**e**). Note the frequency shift toward increased axonal diameters in *Cmtm6*-iKo-mice at both time points PTI. Data are presented as frequency distribution with 0.5 µm bin width; (**d**) *n* = 14814 axons from *n* = 4 control mice and *n* = 20067 axons from *n* = 5 *Cmtm6*-iKO mice; Mean axonal diameter (control+/−*Cmtm6-iKo*) = 3.57 µm + 0.03 µm; *P* = 0.002 by two-sided Kolmogorow–Smirnow test; **e** *n* = 19,025 axons from *n* = 5 control mice and *n* = 15,047 axons from *n* = 4 *Cmtm6*-iKO mice; mean axonal diameter (control+/−*Cmtm6-iKo*) = 4.05 µm + 0.17 µm; *P* = 2.98e^−^^10^ by two-sided Kolmogorow–Smirnow test of frequency distributions. **f**, **g** Quantifications on semi-thin sections shows normal number of myelinated axons in *Cmtm6*-iKo compared to control mice 2 mo PTI (**f**) and 6 mo PTI (**g**). **f** *n* = 4 control and *n* = 5 *Cmtm6*-iKo mice, *P* = 0.2771 (**g**) *n* = 5 control and *n* = 4 *Cmtm6*-iKo mice, *P* = 0.5394; by two-tailed Student’s *t*-test. Data in (**f**) and (**g**) presented as mean ± SD. n.s. = non-significant *P > 0.05; **P < 0.01; ***P < 0.001. Source data see Source Data file.

The results presented thus far were gained with mice, in which *Dhh*^*Cre*^-driven recombination of the *Cmtm6*-gene occurred early in Schwann cell-development, i.e., before the onset of myelination. We thus utilized *Plp*^*CreERT2*^-mice^28^ for tamoxifen-induced recombination in myelinating cells of young adult mice. Indeed, *Cmtm6*^*flox/flox*^;*Plp*^*CreERT2*^-mice injected with tamoxifen at the age of 2 months (termed *Cmtm6*-iKo in the following) displayed increased axonal diameters both 2 and 6 months after tamoxifen injection (PTI) (Fig. 4). CMTM6 thus continues to restrict the growth of axonal diameters after developmental myelination is largely completed in young adult mice.

### Assessment of Remak bundles

Peripheral axons below 1 µm in diameter are ensheathed by non-myelinating Schwann cells in Remak bundles^7^. Developmentally, axons reaching a diameter above this 1 µm threshold are radially sorted out of bundles to be myelinated by individual Schwann cells. Considering that mutations in numerous genes impair radial sorting^7^, we assessed Remak bundle-associated axons in *Cmtm6*-cKo sciatic nerves. Surprisingly, the diameters of these non-myelinated axons were also significantly increased at P9 and, more strikingly, at 2 months (Supplementary Fig. 9a–d). Yet, the number of axons per Remak bundle was unaltered (Supplementary Fig. 9e, f). Importantly, Remak bundles did not comprise axons above the 1 µm threshold diameter (Supplementary Fig. 9g, h), and the frequency of developmentally sorted, promyelinated axons were unchanged (Supplementary Fig. 10c). We note that the diameters of myelinated axons were not yet significantly increased at the early developmental time point of P9 (Supplementary Fig. 10a, b); indeed, significance was reached by 1 month of age (Supplementary Fig. 10e, f). Together, CMTM6 restricts radial growth of both non-myelinated and myelinated axons without interfering with radial sorting or myelin biogenesis.

Expression of CMTM6 can affect the presence of programmed death ligand-1 (PDL1/CD274) at the surface of cells in a tumor environment at least in vitro, a possible mechanism to regulate anti-tumor immunity^29,30^. To test whether CMTM6 regulates CD274 in non-tumorigenic Schwann cells in vivo, we assessed *Cmtm6*-cKo-mice. However, immunoblotting of homogenized peripheral nerves (Supplementary Fig. 11a–c) and immunohistochemistry of cross-sectioned sciatic nerves (Supplementary Fig. 11d) showed unaltered abundance and preferentially abaxonal localization of CD274. *Vice versa*, the abundance of CMTM6 was also unchanged in *Cd274*-Ko sciatic nerves by immunoblot (Supplementary Fig. 11e).

### Increased axonal diameters in *Cmtm6*-cKO;*Mag*-KO mice

**Fig. 5:**
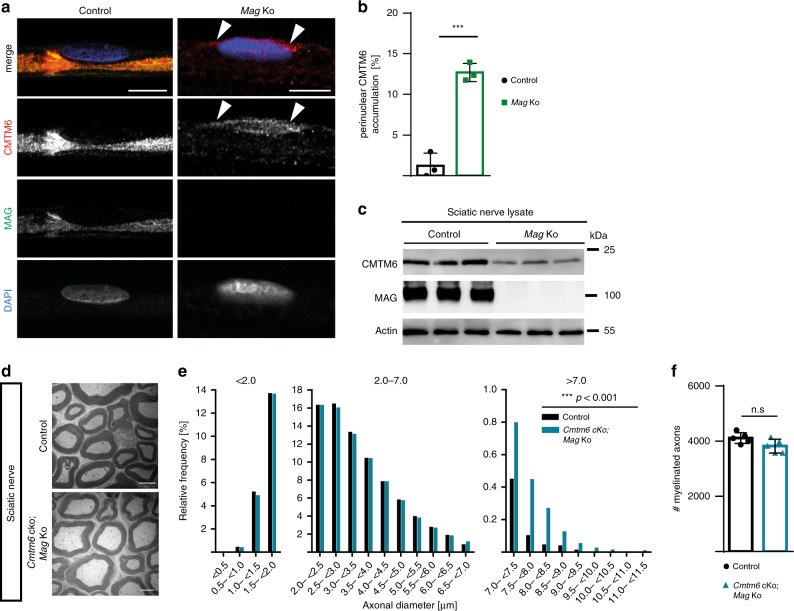
Axonal diameters are increased in the absence of both CMTM6 and MAG. **a**, **b** Immunolabeling of teased fiber preparations from sciatic nerves of 2-mo-old mice and genotype-dependent quantification reveals perinuclear accumulation of CMTM6 (red) when MAG (green) is lacking (indicated by white arrowheads). Nuclei were labeled with DAPI (blue). Scale bar, 10 µm. *n* = 3 mice per genotype; *P* = 0.0005 by two-tailed Student’s *t*-test. **c** Immunoblotting shows decreased abundance of CMTM6 in *Mag*-Ko compared to control sciatic nerves at P75. Blots shows *n* = 3 mice per genotype. **d** Representative electron micrographs of cross-sectioned sciatic nerves show increased axonal diameters in the sciatic nerves of *Cmtm6*-cKo;*Mag*-Ko double-knockout compared to control mice at 2 mo. Scale bar, 2.5 µm. **e** Genotype-dependent quantification reveals frequency distribution shift toward increased diameters of myelinated axons in sciatic nerves of *Cmtm6*-cKo;*Mag*-Ko double-knockout compared to control mice at 2 mo. Data are presented as frequency distribution with 0.5 µm bin width; *n* = 19108 axons from *n* = 5 control mice and *n* = 18003 axons from *n* = 5 *Cmtm6*-cKo;*Mag*-Ko double-knockout mice; Mean axonal diameter (control+/− *Cmtm6-cKo;Mag-Ko double-knockout)* *=* 3.13 µm + 0.09 µm; *P* = 0.0001 by two-sided Kolmogorow–Smirnow test of frequency distributions. **f** Quantitative assessment shows unchanged numbers of myelinated axons in *Cmtm6*-cKo;*Mag*-Ko double-knockout compared to control mice. *n* = 5 per genotype; *P* = 0.0761 by two-tailed Student’s *t*-test. Data in (**b**) and (**f**) presented as mean ± SD. n.s. = non-significant *P < 0.05; **P < 0.01; ***P < 0.001. Source data see Source Data file.

It was previously reported that mice lacking myelin-associated glycoprotein (MAG) have mildly reduced diameters of peripheral axons^14^ and our assessment confirmed this finding (Supplementary Fig. 12). Considering that the two adaxonal Schwann cell molecules that increase (MAG) or restrict (CMTM6) axonal diameters may functionally interact, we assessed their abundance and localization in the absence of the respective other. Abundance and localization of MAG is clearly independent of CMTM6 (Fig. 1i and Supplementary Fig. 3b). Vice versa, however, in *Mag*-Ko-mice CMTM6 displays a mainly perinuclear localization in Schwann cells according to immunolabeling of teased fiber preparations (Fig. 5a, b), and its abundance was strongly reduced by immunoblotting (Fig. 5c). Thus, MAG facilitates normal abundance and localization of CMTM6 in Schwann cells.

**Fig. 6:**
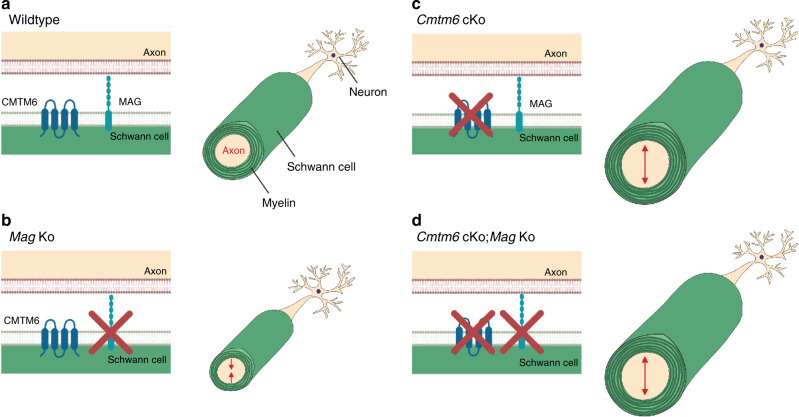
Model of the role of adaxonal myelin proteins CMTM6 and MAG in regulating the diameters of peripheral axons. **a**, **b** Compared to wild-type mice (**a**), the diameters of peripheral axons (light brown) are decreased in the absence of myelin-associated glycoprotein (MAG) (**b**) (see ref. ^14^ and Supplementary Fig. 12), which is specifically expressed by Schwann cells (green). This implies that MAG-dependent signaling by Schwann cells increases axonal diameters^14^. **c** The diameters of peripheral axons are markedly increased (arrows) when Schwann cells lack CMTM6, indicating that Schwann cells utilize CMTM6 to restrict axonal diameters. **d** Diameters of peripheral axons are increased (arrows) when Schwann cells lack both MAG and CMTM6, indicating that loss-of-CMTM6-function overrides loss-of-MAG-function regarding axonal diameters. Schwann cells thus regulate the diameters of peripheral axons via adaxonal surface proteins including MAG and CMTM6. Scheme created by Maria A. Eichel with Biorender.com.

Considering this interaction between MAG and CMTM6, we finally assessed axonal diameters in the absence of both proteins. Strikingly, axonal diameters were increased in *Cmtm6-*cKo;*Mag*-Ko double-knockout mice like in *Cmtm6-*cKo single-knockout mice (Fig. 5d, e). This indicates that loss-of-CMTM6-function overrides loss-of-MAG-function regarding axonal diameters (scheme in Fig. 6). Taken together, Schwann cells regulate the diameters of peripheral axons via adaxonal surface proteins including MAG and CMTM6.

## Discussion

The velocity of impulse propagation along fibers in the nervous system depends on myelination, axonal diameters, nodal features, and internode length^19^. In non-myelinated species, only a few vital reflexes are mediated by giant-diameter axons, probably owing to constraints by space and energy consumption^3^. Indeed, NCV along non-myelinated fibers increases approximately proportional to the square root of the interior axonal diameter^20,21^. Compared to non-myelinated fibers, variation of the diameters of myelinated axons affects NCV much more potently when considering the roughly linear proportionality^19,21,22^. Strikingly, myelin facilitates saltatory conduction^1,2^, thereby accelerating NCV 20–100-fold compared to unmyelinated axons of the same diameter^3^. Theoretically, this would allow more fast-propagating axons with a reduction of required space and metabolic demand. It may thus appear counter-intuitive that so far no myelin-dependent mechanism was identified that restricts axonal diameters. Indeed, the expression of MAG in Schwann cells further increases the diameters of myelinated peripheral axons^14^. Here, we used label-free proteome analysis to assess the constituents of a biochemical fraction from peripheral nerves termed axogliosome-enriched fraction. Utilizing STED microscopy and cryo-immuno electron microscopy, we identified CMTM6 as a constituent of the adaxonal Schwann cell membrane. To our knowledge, CMTM6 is the first myelin protein found to actually restrict axonal diameters. Importantly, deficiency of CMTM6 does not impair the radial sorting of axons, myelin biogenesis, axonal integrity, internode length, or nodal/paranodal parameters. This implies that the function of CMTM6 in Schwann cells is specifically the restriction of axonal diameters.

CMTM6 is a member of the chemokine-like factor-like MARVEL-transmembrane domain-containing (CMTM)-family of proteins^31^. Structurally, CMTM-proteins are predicted to comprise four transmembrane domains, short N- and C-terminal stretches protruding into the cytoplasm and two extracellular loop domains. Its name notwithstanding, CMTM6 does not comprise an apparent chemokine-like domain. Expression of CMTM6 was reported to limit anti-tumor immunity^29,30^ via regulating abundance and localization of programmed death ligand-1 (PDL1/CD274) at the surface of cancer cells, at least in vitro. However, we did not find evidence of CMTM6 influencing CD274 in Schwann cells, at least in non-tumorigenic peripheral nerves in vivo. It will be interesting to elucidate in future work whether CMTM6 in Schwann cells affects the abundance or localization of other proteins, or possibly lipids^32^.

Our finding that *Cmtm6* -cKO nerves display normal *g*-ratios at multiple time points of sciatic nerve development implies that axonal diameters and myelin thickness effectively increase at the same time. We can thus not formally rule out that CMTM6 in Schwann cells may function in *cis* to increase myelin thickness, which, by an unknown indirect mechanism may secondarily enhance radial axonal growth. However, considering the adaxonal localization of CMTM6 and MAG it is likely that Schwann cells regulate axonal diameters directly in trans via a CMTM6-dependent mechanism. In turn, appropriate myelin sheath thickness in *Cmtm6* -cKO nerves is expected to emerge secondary to axonal diameter increase via neuregulin-1 signaling to erbB-receptors^8^. We did not find evidence of altered neurofilament phosphorylation or density in *Cmtm6* -cKO axons, suggesting that other axonal molecules execute Schwann cell-dependent regulation of radial axonal growth. For example, axonal diameters are increased in mice lacking the cytoskeletal regulator α-adducin (ADD1), probably by regulating the diameters of axonal submembrane actin/spectrin rings^33,34^. Though speculative, Schwann cells may restrict secondary axonal growth via axonal actin/spectrin rings. Circumstantially, CMTM6 in the adaxonal Schwann cell membrane displays periodic distribution similar to axonal β2-spectrin.

Given that the number of myelinated axons, internode length and nodal features are unaltered in *Cmtm6* -cKO nerves, the observed increase in SNCV is expected to be due to the shift toward increased axonal diameters. Notably, as measured, NCV is mainly determined by the fibers with the largest diameters^18^. Yet, the normal *g*-ratios in *Cmtm6* cKO nerves indicate that coinciding with the increased axonal diameters, myelin sheath thickness is also appropriately increased. Indeed, thicker myelin sheaths increase the transverse resistance, and thus reduce the transverse capacitance of a fiber, by itself correlating with increased NCV^19,21^. The effects of axonal diameters and myelin thickness on NCV can thus not be experimentally uncoupled in the present model. Conversely, in theoretical calculations and simulation studies usually one parameter is theoretically altered while assuming all other relevant parameters as fixed, ultimately limiting direct correlation with experimental data and biological complexity. Finally, we cannot formally rule out the existence of yet unidentified additional factors that may affect NCV in *Cmtm6* -cKO nerves despite careful morphological and quantitative assessment.

It is of note that increased axonal diameters in *Cmtm6* -cKO mice were observed in four different peripheral nerves, implying that the effect is not limited to a particular nerve type. Yet, among the assessed nerves the shift was most considerable for the phrenic nerve, a vital nerve that controls breathing^35^. Indeed, *Cmtm6* cKO mice displayed anomalous breathing, i.e., the lack of normal breathing pauses. Interestingly, phrenic nerves display a bimodal frequency distribution of axonal diameters in control mice, which was altered toward unimodal, roughly normal distribution in *Cmtm6* cKO mice. We cannot exclude that the effect of CMTM6-deficiency may be somewhat more pronounced for particular axonal sub-populations, e.g., for sensory versus motor fibers. However, it is not straightforward to conclude from bimodal versus unimodal distributions if a different susceptibility of sensory or motor axons exists when considering that bimodal distribution of axonal diameters are observed both in mixed (e.g., phrenic) and non-mixed nerves (e.g., ventral root, a motor nerve) while other mixed nerves do not display bimodal distribution (e.g., sciatic nerve, tail nerve). In the phrenic nerve, the frequency distributions of axonal diameters, as well as the total number of fibers, vary markedly among species. For example, phrenic fibers display bimodal distribution of diameters in cats and dogs^36,37^ but unimodal distribution in rabbits^38^. When comparing young and adult rats, the number of myelinated fibers is roughly similar; however, the unimodal distribution of phrenic fibers in young rats^39^ shifts to bimodal distribution in adult rats^40^. Together, *Cmtm6* -cKO mice display a shift toward larger axonal diameters in multiple types of peripheral nerves and at multiple developmental stages; yet, it is difficult to interpret from the distribution patterns whether particular axonal sub-populations are more susceptible than others.

Expression of MAG^14^ and CMTM6 affect axonal dimensions toward enhancing and restricting radial growth, respectively, suggesting functional interplay of both adaxonal myelin proteins in optimizing nerve function. However, the functional relevance of the expression of MAG and CMTM6 is more complex. For example, MAG-deficiency causes early postnatal loss of motoneurons^41^ and of peripheral axons^42^, whereas *Cmtm6* -cKO mice did not display altered numbers of myelinated axons, at least up to an age of 2 months. Moreover, CMTM6-deficiency does not impair abundance or localization of MAG; vice versa MAG-deficiency leads to reduced abundance of CMTM6 in peripheral nerves coinciding with perinuclear accumulation and probably accelerated turnover in Schwann cells. Yet, axonal diameters are increased when both proteins are lacking, similar to deficiency of only CMTM6. This implies that CMTM6 may affect axonal diameters also if at low abundance and retained in intracellular compartments. Future work will show whether experimentally overexpressing CMTM6 restricts axonal diameters beyond its effect at wild-type levels. We hypothesize that besides MAG and CMTM6 additional, unidentified molecules are involved in the extrinsic regulation of secondary radial axonal growth by Schwann cells. If such molecules exist and are proteins, they are probably comprised in the axogliosome-enriched fraction. Further exploitation of the proteomic dataset may thus lead to their identification and enable characterizing their functional interplay with MAG and CMTM6.

In conclusion, CMTM6 emerges as a key player in the interactions between Schwann cells and axons. We speculate that counteracting the function of CMTM6 may restore the reduced axonal diameters and slowed NCV^18,43^ in rodent models of Charcot–Marie–Tooth spectrum disorders. If confirmed in future preclinical assessment, this may allow developing a rational therapy concept toward functional improvement for neuropathy patients.

## Methods

### Mouse models

Mice lacking expression of MAG (*Mag*^*null*^ mice; also termed *Mag* Ko) or CD274 (*Cd274*^*null*^ mice; also termed *Cd274*-Ko) were previously described^44,45^. Genotypes were determined by genomic PCR. Primers to determine the *Mag* allele were sense 5′-TTGGCGGCGA ATGGGCTGAC, sense 5′-ACGGCAGGGA ATGGAGACAC and antisense 5′-ACCCTGCCGC TGTTTTGGAT, amplifying a 600 bp band for the *Mag*^*null*^ allele and a 300 bp band for the WT allele. Primers to determine the *Cd274* allele were sense 5′-AGAACGGGAG CTGGACCTGCTTGCGTTAG, sense 5′-GCCTTCTTGA CGAGTTCTTC and antisense 5′-ATTGACTTTC AGCGTGATTCGCTTGTAG amplifying a 450 bp band for the *Cd274*^*null*^ allele and a 250 band for the WT allele. Experimental homozygous mutant mice and control littermates were the progeny of heterozygous mice.

Frozen mouse sperm comprising the *Cmtm6*^*tm1a(EUCOMM)Wtsi*^ allele (also termed *Cmtm6*^*lacZ/neo*^) was obtained from the European Mouse Mutant Archive (EMMA, Neuherberg/Munich, Germany) and used for in vitro fertilization, yielding mice harboring the *Cmtm6*^*lacZ/neo*^ allele. Upon interbreeding with mice expressing FLIP recombinase (129S4/SvJaeSor-*Gt(ROSA)26Sor*^*tm1(FLP1)Dym*^*/J*; backcrossed into C57BL/6N) the lacZ/neo cassette was excised in vivo, yielding mice carrying the *Cmtm6*^*tm1c(EUCOMM)Wtsi*^ allele (also termed *Cmtm6*^*flox*^). To inactivate expression of *Cmtm6* in Schwann cells, exon 2 and 3 were excised in vivo upon appropriate interbreedings of *Cmtm6*^*flox*^ mice with mice expressing *Cre* under the control of the *Dhh* promotor^25^. For simplicity, *Cmtm6*^*flox/flox*^*;Dhh*^*Cre*^ mice are also termed *Cmtm6* conditional knockout (*Cmtm6* cKo). To inactivate the expression of *Cmtm6* in adult Schwann cells, *Cmtm6*^*flox/flox*^ mice were interbred with mice expressing tamoxifen-inducible *Cre* recombinase under the control of the *Plp* promotor (*Plp*^*CreERT2*^ mice)^28^. Both *Cmtm6*^*flox/flox*^ (termed control) mice and induced conditional knockout mice (*Cmtm6*^*flox/flox*^*; Plp*^*CreERT2*^; termed *Cmtm6* iKo) were injected tamoxifen intraperitoneally (i.p.) at the age of 8 weeks (1 mg tamoxifen dissolved in 100 µl corn oil per mouse per day) for 10 days with a break of 2 days after the first 5 days of injection (scheme in Fig. 4a; protocol adapted from ref. ^28^). Routine genotyping of *Cmtm6* cKo mice was by genomic PCR using sense primer (5′-GCTGCTGTTT CTCATTGCTG; P1 in scheme in Supplementary Fig. 4a) in combination with antisense primers (5′-TGTGTCAAAC GCTAAGACTCAGA; P2, amplifying a 292 bp band for WT allele and a 450 bp band for *Cmtm6*^*flox*^ allele) and (5′-GAGCTCAGAC CATAACTTCG); P3, amplifying a 350 bp band for recombined *Cmtm6*^*flox*^ allele. PCR genotyping of *Dhh*^*Cre*^ was with primers 5′-CAGCCCGGAC CGACGATGAA and 5′-CCTGCGGAGA TGCCCAATTG, amplifying a 400 bp band. PCR genotyping of *Plp*^*CreERT*^ was with primers 5′-TGGACAGCTG GGACAAAGTAAGC and 5′-CGTTGCATCG ACCGGTAATGCAGGC, amplifying a 250 bp band. To inactivate both *Cmtm6* and *Mag*, appropriate interbreedings of *Cmtm6*^*flox/flox*^*;Dhh*^*Cre*^ and *Mag*^*null*^ mice yielded *Cmtm6*^*flox/flox*^*;Dhh*^*Cre*^;*Mag*^*null*^ mice (also termed *Cmtm6 cKo;Mag Ko*). Experimental mutant mice were analyzed together with littermate controls as far as possible. Mice were bred and kept in the mouse facility of the Max Planck Institute of Experimental Medicine. All animal experiments were performed in accordance with the German animal protection law (TierSchG) and approved by the Niedersächsisches Landesamt für Verbraucherschutz und Lebensmittelsicherheit (LAVES) under license 33.19-42502-04-16/2128.

### Biochemical purification of an axogliosome-enriched fraction

To purify a light-weight membrane fraction enriched for the plasma membrane of peripheral axons and the adaxonal Schwann cell membrane, we adapted protocols originally established for the brain^23,24^. Briefly, for each biological replicate, we pooled the sciatic nerves dissected from ten adult C57BL/6N mice at postnatal day 75 (P75) in centrifugation tubes containing 1.25 M sucrose supplemented with complete protease inhibitor tablets (Roche Diagnostics GmbH, Mannheim, Germany) for homogenization using Polytron PT3000 (Kinemetica, Eschbach, Germany). The nerve lysate was carefully overlaid with first 1 M and then 0.29 M sucrose (see scheme in Supplementary Fig. 1). Floating-up centrifugation was carried out using an XL-70 ultracentrifuge with Optima^TM^ TLX rotor (Beckman Coulter, Krefeld, Germany) at 100,000×*g* for 16 h at 4 °C. Using a Pasteur pipette, we then collected the myelin-enriched fraction at the 0.29 M/1 M interphase and the axogliasome-enriched fraction (AEF) at the 1 M/1.25 M interphase. Two subsequent washing steps and osmotic shocks were performed with both fractions. To this aim, the interface was collected, 1 ml ice-cold _dd_H_2_O was added and vortexed. Then, the tubes were filled up with ice-cold _dd_H_2_O up to 0.5 cm below the tube edge and centrifuged for 15 min at 100,000 × *g*. After centrifugation, the supernatant was discarded carefully and the remaining fluid was removed using paper tissue. This washing step was repeated once^46^. The AEF-pellet was taken up in 50 µl 10× TBS with protease inhibitor (Roche), snap-frozen, and stored at −80 °C. The myelin-enriched fraction pellet was taken up in 100 µl 10× TBS including protease inhibitor (Roche), snap-frozen and stored at −80 °C. Protein concentrations were determined using the DC protein assay (BioRad, Munich, Germany). For proteome analyses, the AEF was purified from nine pools of sciatic nerves, considered as nine biological replicates. The immunoblot in Fig. 1b displays the analysis of nerve lysate, myelin-enriched fraction, and AEF of two biological replicates purified in the same experiment.

### Proteome analysis

Isolated axogliasome-enriched fractions (AEF) were subjected to proteolytic digestion using filter-aided sample preparation (FASP) as previously described in detail^47,48^. AEF purified from nine pools of sciatic nerves considered as nine biological replicates were assessed; of each biological replicate three technical replicates were analyzed. Briefly, samples (corresponding to 20 µg of total protein) were loaded onto spin filter columns (Nanosep centrifugal devices, 30 kDa MWCO; Pall, Port Washington, NY) and detergents were removed washing the samples three times with a buffer containing 8 M urea. Proteins were reduced using dithiothreitol (DDT) and alkylated with iodoacetamide (IAA). Excess IAA was quenched by the addition of DTT. Afterward, the membrane was washed three times with 50 mM NH_4_HCO_3_ and proteins were digested overnight at 37 °C using trypsin (Trypsin Gold, Promega, Madison, WI) at an enzyme-to-protein ratio of 1:50 (w/w). After proteolytic digestion, peptides were recovered by centrifugation and two additional washes with 50 mM NH_4_HCO_3_. Flow-throughs were combined and samples were acidified with trifluoroacetic acid (TFA) to a final concentration of 1% (v/v) TFA. After lyophilization, purified peptides were reconstituted in 0.1% (v/v) formic acid (FA) for Liquid Chromatography–Mass Spectrometry (LC–MS) analysis.

LC separation of peptides was performed using a nanoAcquity UPLC system (Waters Corporation) equipped with a 75 μm × 250 mm C18 HSS-T3 1.8 μm reverse-phase column. Peptides corresponding to ~200 ng protein were loaded onto the column using direct injection mode. For reverse-phase separation of peptides, two mobile phases were used. Mobile phase A contained 0.1% FA in water and mobile phase B 0.1% FA in acetonitrile (ACN). Tryptic peptides were separated at 55 °C using a gradient of 5–40% mobile phase B over 90 min at a flow rate of 300 nl/min. MS analysis was performed on a nano-ESI-Q-TOF mass spectrometer (Waters Corporation Synapt G2-S HDMS) equipped with an ion mobility separation (IMS) device. LC–MS data were collected in DIA (data-independent acquisition) mode using MS^E^ combined with IMS (UDMS^E^)^48,49^. [Glu^1^]-fibrinopeptide (100 fmol/µl) served as lock mass and was sampled every 30 s into the mass spectrometer via the reference sprayer of the NanoLockSpray source.

LC–MS DIA raw data were processed and searched with ProteinLynx Global SERVER (PLGS) (version 3.02, Waters Corporation) against a custom compiled database containing UniProtKB/SwissProt entries of the mouse reference proteomes (entries: 16,580) as well as common contaminants. We applied the following search criteria: (i) Trypsin as digestion enzyme allowing up to two missed cleavages, (ii) carbamidomethyl cysteine was defined as fixed and methionine oxidation as variable modification. (iii) Peptides had to have a minimum length of six amino acids. The false discovery rate (FDR) for peptide and protein identification was evaluated searching a reversed database and set to 1% for database search in PLGS. Post-processing and label-free quantification analysis was performed using ISOQuant version 1.8^48,49^. For each protein, absolute in-sample amounts were estimated using TOP3 quantification^50^. Based on the quantitative information derived from the TOP3 approach, we calculated the relative amount of each protein with respect to the sum over all detected proteins [ppm: parts per million (w/w) of total protein]. To be included in the final list, peptides had to be identified in at least three biological replicates (two peptides/protein). Moreover, only peptides with a PLGS identification score equal or above 5.5 were considered.

### Immunoblotting

For immunoblotting, either biochemically purified fractions as described above were used or animals were killed by cervical dislocation, respective tissue was dissected, snap-frozen on dry ice, and stored at −80 °C until further processing. Age of mice and tissue are specified in the figure legends. Tissue was homogenized in 400 µl pre-cooled RIPA Buffer (1× TBS, 1 mM EDTA, 0.5% Sodium deoxycholate, 1.0% Triton X-100) containing Complete Mini protease inhibitor (Roche Diagnostics GmbH, Mannheim, Germany) using Precellys 24 (Peqlab, Erlangen, Germany) at 5000 rpm for 3 × 10 s + 10 s break. Lysates were kept on ice for 15 min and if not fully homogenized the second step of homogenization was performed. Lysates were then centrifuged 5 min at 13,000 rpm at 4 °C, the supernatant was transferred into a new tube and stored at −80 °C. To measure protein concentrations of samples the BioRad DC Protein Assay kit was used following manufacturer’s instruction and optical density was measured at 650 nm using the Eon™ High Performance Microplate Spectrophotometer (BioTek, Vermont, USA). Samples were further diluted in 1× SDS sample buffer and 5% β-mercaptoethanol to denature proteins. As an exception, when detecting MAG, we used non reduced conditions without β-mercaptoethanol. Samples were kept at −20 °C. Before usage samples were heated for 10 min at 40 °C. Protein separation was performed by SDS-PAGE (10–15% acrylamid gels) using Mini-PROTEAN Handcast system (BioRad, Munich, Germany). Between 5 and 25 µg of sample (depending on protein) were loaded onto the gel as well as 5 µl pre-stained protein ladder (PageRuler™, ThermoFischer Scientific) and were separated by constant current (200 V) for 1 h. For immunodetection, proteins were transferred to a PVDF membrane (GE Healthcare, Buckinghamshire, UK; Cat#10600023) using a Novex® Semi-Dry Blotter (Invitrogen, Karlsruhe, Germany). Beforehand, membranes were activated 1 min with 100% ethanol and washed two times in _dd_H_2_O. Proteins were transferred at 20 V for 45 min using a BioRad power supply. After blotting, membranes were incubated in blocking buffer (5% non-fat dry milk in 1× TBS containing 0.05% Tween-20 (Promega, Fitchburg, USA)) for 1 h at RT. Primary antibodies were diluted in 5 ml blocking buffer and incubated overnight at 4 °C on a horizontal rotor. Afterward membranes were washed three times with 1× TBS-T, incubated with horseradish peroxidase (HRP)-coupled secondary antibodies (diluted in blocking buffer) for 1 h at RT and again washed three times. Bands were visualized using enhanced chemiluminescent detection (ECL) according to the manufacturer’s instructions (Western Lightning® Plus-ECL or SuperSignal™ West Femto Maximum Sensitive Subrate; ThermoFischer Scientific, St. Leon-Rot, Germany). Immunoblots were scanned using ECL Chemostar (Intas Science Imaging, Göttingen, Germany)^51,52^.

Antibodies were specific for CMTM6 (OriGene, Cat# TA322304*,* 1:500), MAG (clone 513; Chemicon, Cat# MAB1567; 1:500), cyclic nucleotide phosphodiesterase (CNP; Sigma, Cat# C5922; 1:1000), ATPaseα1 (abcam, Cat# ab7671, 1:2500), actin (Chemicon, Cat# MAB1501, 1:2000), alpha-Tubulin (Sigma, Cat# SAB2102603, 1:5000), NFASC155 (kindly provided by P. Brophy^53^; 1:1000); MPZ (kindly provided by J. Archelos-Garcia^54^; 1:2000), peripheral myelin protein 2 (PMP2, PTG, Cat# 12717-1-AP, 1:1000); neurofilament heavy chain (NEFH, SMI32; Covance, Cat# SMI32-P, 1:500), phosphorylated-NEFH (SMI31; Covance, Cat# SMI31-P, 1:500), CD274/PDL1 (abcam, Cat# ab213480, 1:500). Secondary HRP-coupled antibodies were HRP-goat anti-mouse IgG (Dianova, Cat# 115-03-003) or HRP-goat anti-rabbit IgG (Dianova, Cat# 111-035-003) (Dianova, Hamburg, Germany) in a dilution of 1:10,000. Bands were visualized using enhanced chemiluminescent detection (ECL) according to the manufacturer’s instructions (Western Lightning® Plus-ECL or SuperSignal™ West Femto Maximum Sensitive Subrate; ThermoFischer Scientific, St. Leon-Rot, Germany). Immunoblots were scanned using ECL Chemostar (Intas Science Imaging, Göttingen, Germany). Quantifications for NEFH- and phosphorylated-NEFH abundances (Supplementary Fig. 8c–f) were performed by measuring band intensity using ImageJ software (https://imagej.nih.gov/ij/) and normalizing to band intensities of ATP1a1 detected on the same membrane (*n* = 3). In general, immunoblots were replicated three times with an *n* = 3 per genotype whenever possible.

### Quantitative real-time PCR

mRNA abundances were determined by qRT-PCR (Supplementary Fig. 4c) using sciatic nerves of 8-week-old male mice of the indicated genotypes. Frozen sciatic nerves were homogenized in Trizol (Life Technologies, ThermoFischer Scientific, St. Leon-Rot, Germany) and RNA was extracted and purified using RNeasy Miniprep kit (Qiagen, Portland, USA). Integrity of purified RNA was confirmed using the Agilent RNA 6000 Nano kit and Agilent 2100 Bioanalyser (Agilent Technologies, Santa Clara, California, USA). cDNA was synthesized using random nonamer primers and SuperScript III RNA H Reverse Transcriptase (Invitrogen, Karlsruhe, Germany). A pipetting robot epMotion 5075 (Eppendorf, Hamburg, Germany) was used for pipetting samples, and Quantitative RT-PCR was performed using Power SYBR Green PCR Master Mix (Promega, Fitchburg, USA) and Light Cycler 480II (Roche Diagnostics GmbH, Mannheim, Germany)^55^. The abundance of mRNAs was analyzed in relation to the mean of the standards *Rps13* and *Ube2l3*, which did not differ between genotypes. Statistical analysis was performed in GraphPad Prism 6.0. Primers were specific for *Cmtm3* (forward 5′-GAGGACACCA CGTAGCAGATG, reverse 5′-GAGGACACCA CGTAGCAGATG), *Cmtm4* (forward 5′-GAGGATCCCC CAGATCAACT, reverse 5′-GGCGATAAAG AAAAAGAAAGTGC), *Cmtm5* (forward 5′-TTCCTGTCTT CCCTCAAAGG, reverse 5′-GCCGTGAAGC AAATGAAGAT), *Cmtm6* (forward 5′-GATACTGGAA AAGTCAAGTCATCG, reverse 5′-AATGGGTGGA GACAAAAATGA), *Cmtm7* (forward 5′-TCGCCTCCAT AGTGATAGCC, reverse 5′-CTCGCTAGGC AGAGGAAGC), *Cmtm8* (forward 5′-CAGAGAAGGA AGGGCACAAC, reverse 5′-TGACCAGGAA GGCAAAGAAC), *Rps13* (forward 5′-CGAAAGCACC TTGAGAGGAA, reverse 5′-TTCCAATTAGGTGGGAGCAC), and *Ube2l3* (forward 5′-AGCAGCACCA GATCCAAGAT, reverse 5′-CACATTTGCG GATCTCTTCA). Four biological replicates with four technical replicates each were used.

### STED nanoscopy

Sciatic nerves were fixed in 4% PFA for 45 min at 4 °C, permeabilized in ice-cold methanol for 20 min and rehydrated in PBS. Free-floating partially teased fibers were blocked for 45 min in PBS containing 1% BSA. Both primary and secondary antibody incubations were performed for 1–2 h at RT or overnight at 4 °C in PBS supplemented with 0.05% Triton X-100. After each step, samples were washed three times for 10 min in PBS with 0.05% Triton X-100. Finally, samples were completely teased on a coverslip and embedded in a mounting media containing 9% [w/v] Mowiol (Sigma Aldrich, Darmstadt, Germany) supplemented with DABCO (Sigma Aldrich, Darmstadt, Germany). For preparing cross sections, immunolabeled nerves were teased on a coverslip and embedded in melamine resin following a controlled temperature regime (24 h at RT, 24 h at 40 °C, and 48 h at 60 °C). Melamine resin was prepared by dissolving 48 mg of the resin catalyzer *p*-toluensulfonic acid monohydrate (Sigma Aldrich, Darmstadt, Germany) in 0.576 ml distilled water and then adding 1344 g of 2,4,6-Tris[bis(methoxymethyl)amino]-1,3,5-triazine (TCI, Tokyo, Japan)^56^. After complete polymerization, sections of 200–300 nm thickness were cut using an ultramicrotome (EM UC6, Leica Microsystems, Wetzlar, Germany). The slices were directly transferred onto glass coverslips and embedded in mounting media. Primary antibodies were specific for CMTM6 (OriGene, Cat# TA322304*,* 1:100) and betaII-spectrin (BD Biosciences, San Jose, United States, Cat# 612563, 1:200). Secondary antibodies (sheep anti-mouse, Dianova, Hamburg, Germany, Cat# 515-005-003; goat anti-rabbit, Dianova, Cat# 111-005-003) were labeled with STAR580 (Abberior, Göttingen, Germany, Cat# ST580-0002) or STAR635P (Abberior, Cat# ST635P-002) and used at 1:100 dilution.

Imaging was performed on a two-color Abberior STED 775 QUAD scanning microscope (Abberior Instruments GmbH, Göttingen, Germany) equipped with an Olympus IX83 microscope stand and an Olympus UPlanSApo ×100/1.4 Oil lens. Excitation was achieved with 488, 561, and 640 nm pulsed lasers, while for depletion pulsed lasers at 775 and 595 nm are available. Images were processed and visualized using the ImSpector software package (Max-Planck Innovation; version 14.1-16.1) and ImageJ (imagej.nih.gov/ij; version 1.50b). Smoothing was performed using a 1-pixel low-pass Gaussian filter within the software ImSpector. Brightness and contrast were applied uniformly to all parts of the images. Line profiles were measured along a 3-pixel wide line. For autocorrelation analyses, the region of interest was cropped and rotated to align the vertical axis of the correlation with the axon orientation, maintaining the pixel size. The correlation was calculated with the Matlab R2015b function ‘xcorr2’, beforehand subtracting the average of the image to avoid an uninformative additive triangular signal in the correlation. The profile along the vertical axis of the autocorrelation image was examined for periodicities.

### Immunohistochemistry

For immunolabeling of teased fiber preparations, sciatic nerves dissected from mice of the indicated genotypes at 8–10 weeks were transferred into ice-cold PBS and processed as described below. Using two fine forceps (Dumont No. 5) the epineurium was removed and small nerve pieces were transferred onto a new coverslip. By pulling the fiber bundles carefully apart with both forceps, axons were separated from each other. Slides were dried and stored at −20 °C. For immunolabeling, teased fiber preparations were incubated in 4% PFA for 5 min, 100% methanol for 5 min, 3 × 5 min PBS and 1 h blocking buffer (PBS, 10% horse serum and 0.1% Tween-20) at RT consecutively. Primary antibodies were applied in blocking buffer overnight at 4 °C. Samples were washed again in PBS 3 × 5 min, secondary antibodies diluted in blocking buffer (1:1000) were applied for 1 h at RT and afterward samples were washed with PBS 3 × 5 min and 2 × 30 s in _dd_H2O before being mounted using Aqua-Poly/Mount (Polysciences, Eppelheim, Germany)^57^. Antibodies were specific for MAG (clone 513; Chemicon, Cat# MAB1567; 1:50), CMTM6 (OriGene, Cat# TA322304*,* 1:200), CASPR (Neuromabs, Cat# clone K65/35, 1:500), Nav1.6 (Almonelabs, Cat# ASC-009, 1:500) or CD274/PDL1 (abcam, Cat# ab213480, 1:500). Secondary antibodies were donkey α-mouse-Alexa488 (Invitrogen, Cat# A21206; 1:1000), donkey α-rabbit-Alexa488 (Invitrogen, Cat# A21206; 1:1000), donkey α-mouse-Alexa555 (Invitrogen, Cat# A21202; 1:1000), and donkey α-rabbit-Alexa555 (Invitrogen, Cat# A21202; 1:1000). For representative images, slides were imaged using the confocal microscope Leica SP5. The signal was collected with the objective HCX PL APO lambda blue 63.0.×1.20. DAPI staining was excited with 405 nm and collected between 417 and 480 nm. To excite the Alexa488 fluorophore, an Argon laser with the excitation of 488 nm was used and the emission was set to 500–560 nm. Alexa555 was excited by using the DPSS561 at an excitation of 561 nm and the emission was set to 573–630 nm. To export and process the images LAS AF lite and Adobe Photoshop were used. For quantification on sciatic nerve teased fiber preparations, images were obtained randomly at 10× or ×40 magnification using Axio Observer Z2 (Zeiss) and, if required, stitched using Zeiss Zen2011. For assessment of nodes of Ranvier, the diameter and length of each Nav1.6 positive node was measured and the overall mean per animal was calculated (Supplementary Fig. 7). For paranodes (Supplementary Fig. 7), the diameter and length of both CASPR positive paranodes besides a Nav1.6 positive node was measured the mean of both paranodes beside one node and the overall mean per animal was calculated. For internodal length (Supplementary Fig. 7), the distance between one Nav1.6 positive node and the next was measured using ImageJ software^58^.

To visualize cells with *Cmtm6* gene activity (Fig. 1h), lacZ immunohistochemistry was performed on sciatic nerve teased fibers of heterozygous *Cmtm6*^*LacZ*/neo^ mice. Slides were incubated with X-gal (1.2 mg X-gal per ml, 5 mM potassium ferricyanide, 5 mM potassium ferrocyanide and 2 mM MgCl_2_ in PBS) solution at 37 °C for 2–3.5 h in the dark. Thereafter, they were rinsed with PBS and mounted using Aqua-Poly/Mount (Polysciences, Eppelheim, Germany). Images were captured at ×40 magnification (Zeiss AxioImager Z1).

### Electron microscopy

For immunogold labeling of cryosections^51,55^, cross-sectioned sciatic nerves from 2-month-old WT mice were used. Antibodies were specific for CMTM6 (OriGene, Cat# TA322304; 1:100). Samples were examined using EM912AB-Omega (Zeiss, Oberkochen, Germany) coupled to a wide-angled dual-speed 2k CCD-camera (TRS, Moorenweis, Germany).

For conventional transmission electron microcopy (TEM) nerves were dissected from respective mice and postfixed in Karlsson–Schultz fixative (4% PFA, 2.5% glutaraldehyde in 0.1 M phosphate buffer) solution at 4 °C until further processing. Samples were embedded in epoxy resin (Serva). For transmission electron microscopy, ultrathin section (50 nm) were cut using a PTPC Powertome Ultramicrotom (RMC, Tuscon Arizona, USA) and collected on formvar polyvinyl coated double-sized slot grids (AGAR scientific, Essex, UK). Ultrathin sections were contrasted with UranyLess (Electron Microscopy Science, Hatfield, Panama) for 15–30 min and washed 6× with _dd_H2O^57,59^. For quantitative analyses, 8–20 random, non-overlapping images were taken at a magnification of ×3000 (sciatic nerves, dorsal roots), ×4000 (phrenic nerves), or ×7000 (Remak bundles). *g*-ratio, axonal degeneration, number of non-myelinated axons, and Remak bundles were assessed using ImageJ (Fiji) with *n* = 3–5. *g*-ratios were measured on electron micrographs; *g*-ratios were calculated as the ratio between axonal Feret diameter and Feret diameter of the corresponding myelin sheath^57,60^. As an exception, for assessment of *g*-ratios in ventral tail nerves, we used a randomized grid on light-microscopic semi-thin sections imaged at ×100 magnification (190 axons per nerve). Every myelin sheath lacking an identifiable axon or containing a degenerating-appearing axon according to the presence of tubovesicular structures and amorphous cytoplasm or a high density of cytoskeletal elements was counted as axonal degeneration. For the analysis of Remak bundles, the circumference of each unmyelinated axon within a bundle was measured and the number of axons per bundle was counted. At least 200 axons per animal were assessed. Axons smaller than 0.2 µm could not be assessed using the obtained images. For the number of promyelinated axons, only axons larger than 1 µm were measured, counted, and set in relation to the overall number of axons in the quantified area. To measure neurofilament density (Supplementary Fig. 8a-b), at least 30 images of cross-sectioned, non-angled sciatic nerve axons were obtained at a magnification of ×6300 using the EM912AB-Omega (Zeiss, Oberkochen, Germany). A 0.2 µm^2^ grid was overlaid and the number of neurofilaments within a minimum of 3 grids/axon were counted using ImageJ (Fiji). The mean of the neurofilament number per axon was calculated. In total, 30 axons per mouse with *n* = 3 were quantified. All quantifications were performed blinded to the genotype.

### Histology, semi-thin sections, and axonal diameter determination

Semi-thin (500 nm) cross sections of epon embedded nerves and tissue of indicated genotypes and ages were obtained using a PTPC Powertome Ultramicrotom (RMC, Tuscon Arizona, USA), transferred to a glass slide, dried on a warm plate (60 °C) and stained by applying methylene blue/azur II (1:1) for ~1 min followed by 3× washing with _dd_H2O. Images were acquired at ×100 magnification using a bright-field light microscope (Zeiss AxioImager Z1; coupled to Zeiss AxioCam MRc camera; controlled and stitched by Zeiss Zen 1.0 software). Axonal diameters were semi-automatically quantified using the ROI-based analyze particle function of ImageJ (Fiji) followed by visual inspection. For axonal diameters and *g*-ratio analysis in the tail one of the two caudal ventral nerves was imaged and analyzed. The dissected and analyzed area was located ~50 mm distally from the tail base. Myelinated axons that escaped automated quantification were manually added to include all myelinated axons per nerve. Schwann cell (SC) nuclei were quantified with SC nuclei in close contact to an axon in a 1:1 ratio. Other nuclei in proximity to other cells (mainly macrophages and fibroblasts) and away from axons were not considered^57^. All quantifications were performed blinded to the genotype.

### Nerve conduction velocity measurement

Standard electroneurography (Fig. 3a, b) was performed on mice of indicated genotypes at P75 using a Toennies Neuroscreen® (Jaeger, Joechsberg, Germany)^61^. Briefly, mice were anesthetized with intraperitoneal (i.p.) injection of Ketaminehydrochloride/Xylazinhydrochloride (100 mg/kg BW/8 mg/kg BW). For sensory nerve conduction measurements, a pair of fine steel needle recording electrodes (Schuler Medizintechnik, Freiburg, Germany) was placed upright at the tail base on either side of the tail followed by two proximal stimulation electrodes exactly 50 mm distally. The ground electrode was placed subcutaneously between the pairs. Increasing current pulses were delivered until supramaximal stimulation was achieved and the compound nerve action potential (SNAP) reached a plateau. SNAP measurements were averaged over 20 stimuli. The sensory nerve conduction velocities (sNCV) were calculated from sensory action potential latency measurements over the 50 mm distance.

### Behavioral analysis

All phenotypical analyses were performed by the same investigator blinded toward genotypes using 8–10-week-old mice. To assess motor capabilities (Fig. 3d), mice were placed on a metal grid (1 cm grid size) and allowed to run a distance of 2 m while being videotaped. The number of fore-and hindlimb slips were assessed on a slow-motion video. Assessment was performed one mouse at a time, once per mouse and without prior habituation to the grid. To assess sensory reaction (Fig. 3c), mice were placed on a hot plate (Leica HI 1220; Nussloch, Germany) which was heated to constant 52 °C and surrounded by a clear acrylic cage (open top). A timer was started once mice were placed on the hot plate and the time until mice respond with either licking or retracting one of their hindlimbs was stopped and measured as retraction latency. Afterward mice were immediately removed from the hot plate and placed back in the home cage. Assessment was performed one mouse at a time, once per mouse, and without prior habituation to the hot plate^62^. All behavioral experiments and analyses were performed blinded to the genotype.

### Plethysmography

Breathing was analyzed by unrestrained whole-body-plethysmography (Fig. 3e)^63^. Mice were placed in a plexiglass chamber and could habituate for at least 12 min. Breathing cycles from a subsequent period of 3 min were analyzed. Since mice were allowed to explore the chamber freely, some pressure changes resulted from sniffing. During analysis, we did not discriminate between respiratory cycles associated to different types of behavior. The chamber was used in the flow-through configuration with a negative bias flow of 150 ml/min introduced by a CO_2_/O_2_ sensor (ADinstruments, Sydney, Australia)^64^. Pressure difference between the recording chamber and a reference chamber were captured by a DP103-12 pressure transducer (Validyne Engineering, Northridge, CA, United States) and passed through a sine wave carrier demodulator (CD-15, Validyne Engineering) for digitization (100 kHz sampling rate) with an analog-digital interface (PowerLab/4 s) and LabChart-software (ADInstruments; version 8.1.13). The signal was smoothed (averaging; 50 samples) and offline band pass filtered (3–20 Hz) to remove noise and movement artifacts. The peak detection module of LabChart was used to identify peaks of inspiratory flow. The respiratory rate was calculated as the reciprocal of the averaged peak-to-peak interval using Excel (Microsoft). Intervals longer than 750 ms were considered as breathing pauses, and the number per minute was calculated. Plethysmography and analyses were done blinded to the genotype.

### Statistics and reproducibility

Statistical analyses were mainly performed in GraphPad Prism (GraphPad Software, Inc., San Diego, United States). Data are mainly shown as bar graphs or dot plots as mean ±SD (error bars). Data distribution was assumed to be normal but was not formally tested, except for Plethysmography data. Sample sizes were not predetermined but are as commonly used in the field. Exact sample size/number of mice is mostly shown in the figures and indicated in the figure legends. Outlier tests were performed on all data except axonal diameter values using GraphPad (https://www.graphpad.com/quickcalcs/Grubbs1.cfm). Only for nerve conduction velocity measurement one outlier was found in the control group; no further outliers were identified. For comparing two groups, unpaired two-tailed Student’s *t*-test was applied. For qRT-PCR (Supplementary Fig. 3c), two-way analysis of variance (ANOVA) with Sidak’s multiple comparisons test was performed. Since data of the breathing pauses (Fig. 3e) are not normally distributed, the nonparametric two-tailed Mann–Whitney rank-sum test was applied. We used RStudio (https://www.rstudio.com/, Version 3.4.1) to statistically assess relative frequency distributions of axonal diameters. The script has been deposited at https://github.com/MariaEichel/FrequencyDistributions.git. Briefly, data were used in data frames and a simple linear model was applied. The two-tailed Kolmogorow–Smirnow test was used to assess the shift in the frequency distributions of axonal diameters between two groups independent of bin size, but was only applied if prior two-tailed Student’s *t*-test on the overall distributions gave a significant result. Visualization of axonal diameter frequency distributions was performed by using GraphPad Prism. All measured axonal diameter values per animal were grouped respective to their genotype. Built-in analysis of frequency distribution in % was performed using a bin width of 0.5 or 0.1 µm and binning each replicate. A *P*-value of <0.05 was considered significant in all tests. Significance levels are represented as n.s. = non-significant, *P < 0.05, **P < 0.01, ***P < 0.001 with exact *P*-values given in the figure legends.

Representative electron micrographs (Figs. 2a–d, 4b, c, 5d and Supplementary Figs. 5a, 7a, 8a, 9a, b, 10a, e, 12a) were chosen from the images obtained from 3–5 different biological replicates per genotype. The electron micrograph of immunogold labeling (Fig. 1g) was selected from 20 images from two independent experiments. For immunoblots (Fig. 1b, f and Supplementary Fig. 11e) representative images of 3–5 independent experiments are shown. STED microscopy data underlying Fig. 1c–e represent the results of three independent experiments, while the STED image in Supplementary Fig. 3a represents the results of 20 lines profiles derived from seven independent fields of view of one experiment. For fluorescence labeling, lacZ histochemistry, and confocal microscopy (Figs. 1g, h, 5a and Supplementary Figs. 3b, 7b, 11d) representative images were chosen from three independent experiments.

### Reporting summary

Further information on research design is available in the Nature Research Reporting Summary linked to this article.

## Supplementary information

Supplementary Information

Description of Additional Supplementary Files

Supplementary Data 1

Reporting Summary

## Data Availability

The source data as well as original immunoblot scans underlying Figs. 1–5 and Supplementary Figs. 4–12 are provided as a Source Data File. The mass spectrometry data are provided as Supplementary Data 1. STED microscopy data underlying Fig. 1c–e and Supplementary Fig. 3 are available from the corresponding author on reasonable request. Proteome raw data were processed and searched against a custom compiled database containing UniProtKB/SwissProt entries of the mouse reference proteomes (https://www.uniprot.org/statistics/Swiss-Prot). Proteomic data are provided as Supplementary Data 1. Source data are provided with this paper.

## Source data

Source Data
