# Supplementary material for: CMTM6 expressed on the adaxonal Schwann cell surface restricts axonal diameters in peripheral nerves
Source: Nat Commun. 2020 Sep 9;11:4514. doi: 10.1038/s41467-020-18172-7 (PMC7481192; doi:10.1038/s41467-020-18172-7)
Supplement: Supplementary file 1 — Supplementary Information [file 41467_2020_18172_MOESM1_ESM.pdf]

Supplementary Information

CMTM6 expressed on the adaxonal Schwann cell surface restricts axonal diameters in peripheral nerves

Eichel et al.

Supplementary Figures 1- 12

Supplementary Data 1

Reporting Summary

Source Data file

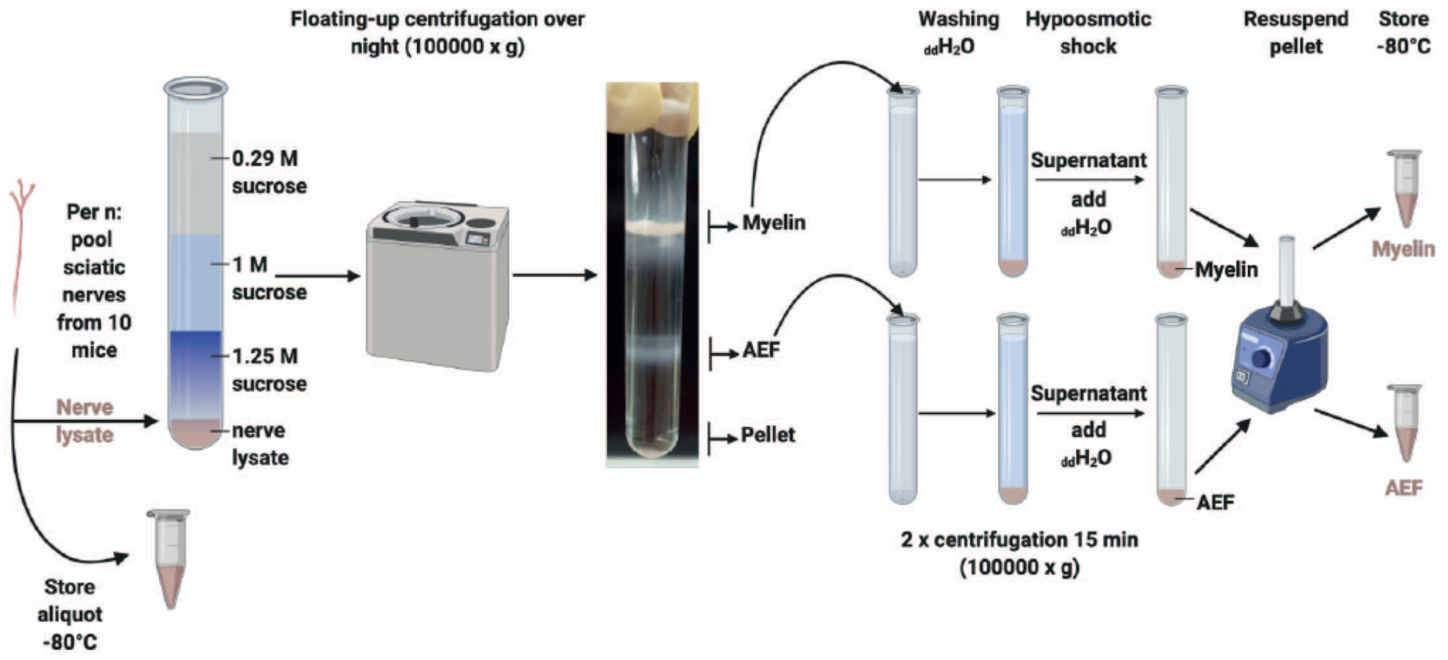

Supplementary Fig.1. Schemes illustrating the biochemical purification of the axogliasome-enriched fraction (AEF) from sciatic nerves

Scheme illustrating the biochemical purification of light-weight membrane fractions enriched for axogliasome or myelin. Sciatic nerve lysates of 10 pooled wild-type mice were overlaid with a sucrose gradient for floating-up ultragradient centrifugation. Subsequent washing and osmotic shocks were performed with both fractions resulting in purified myelin and axogliosome-enriched fractions. For details see Methods. For proteomic assessment of the AEF fraction see Supplementary Table 1, Fig. 1a, Supplementary Fig. 2. Scheme created by Maria A. Eichel with Biorender.com.

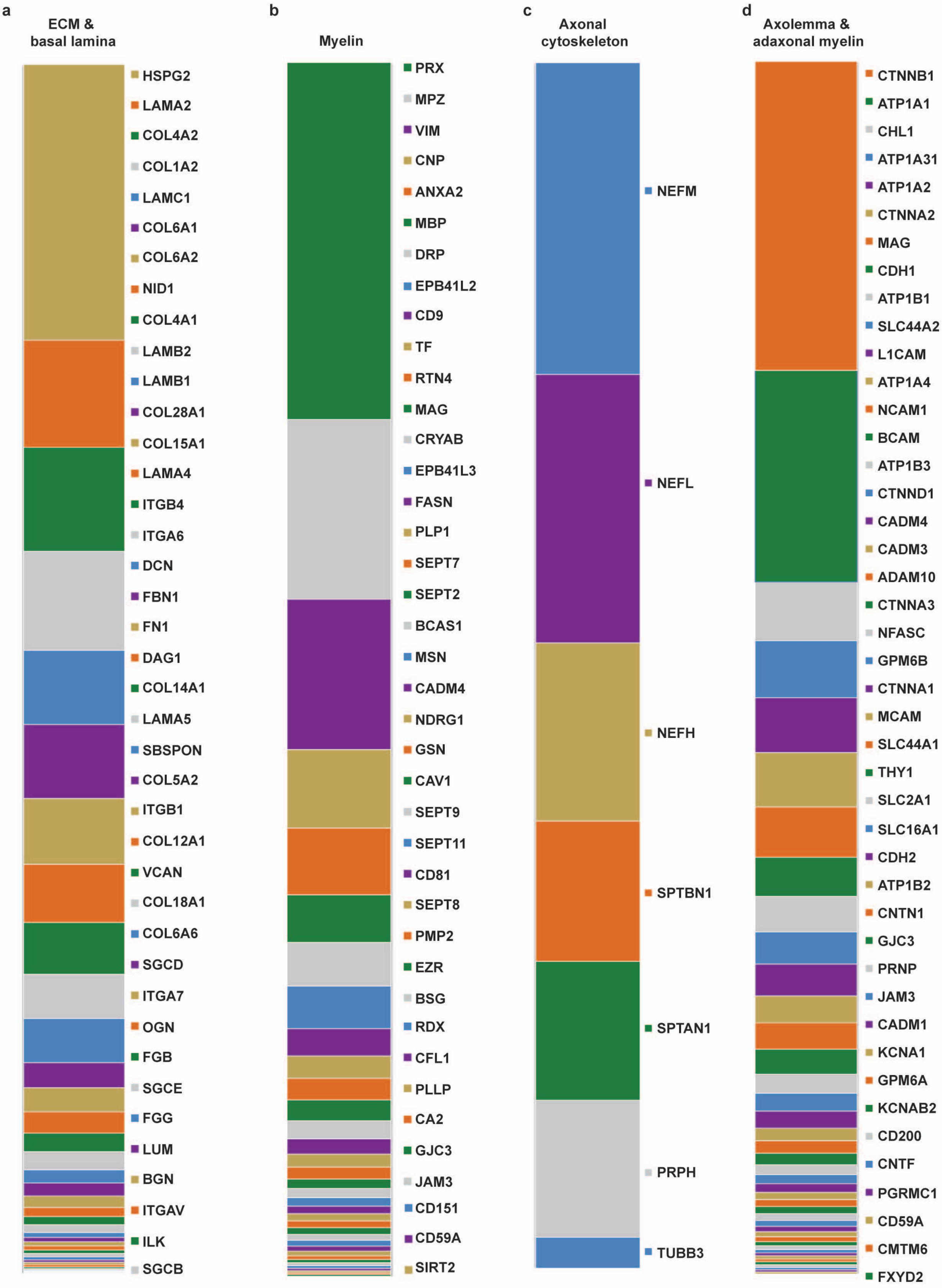

Supplementary Fig. 2. Quantitative proteome analysis shows that the axogliasome-enriched fraction (AEF) purified from sciatic nerves, additional to axolemma and adaxonal myelin, also comprises axonal cytoskeleton, compact myelin, extracellular matrix (ECM) and basal lamina.
Bar charts showing selected proteins of ECM and basal lamina **(a),** myelin **(b)**, axonal cytoskeleton **(c)**, as well as axolemma and adaxonal myelin (d) identified by label-free quantitative mass spectrometry in the axogliasome-enriched fraction (AEF) biochemically purified from sciatic nerves of adult wild-type mice. For pie chart representing the entire AEF see Fig. 1a. For exact values and the entire proteome dataset see Supplementary Table 1. Note that CMTM6 was identified a novel low-abundance constituent of the adaxonal myelin membrane.

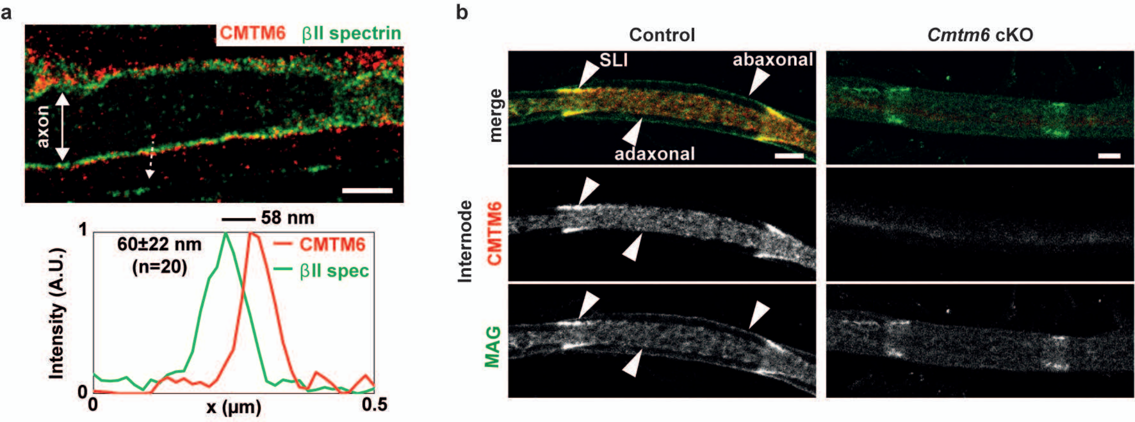

Supplementary Fig. 3. Adaxonal localization of CMTM6 and absence in *Cmtm6*-cKo mice.

(a) STED imaging of longitudinal thin sections (250 nm) of sciatic nerves (P60-70) stained with CMTM6 (red) and βII spectrin (green) show that CMTM6 localizes outside of the axonal cytoskeletal protein βII spectrin. White arrow indicates the location and direction on which the line profile of intensities shown in the lower panels has been drawn. Numbers indicate the distance between the center of the intensities peaks, calculated with a Gaussian fitting. Average distance and related standard deviation between the center of the CMTM6 and βII spectrin peaks indicated in the lower panel of has been calculated for 20 line profiles from 7 images (mice P60-140). All images are smoothed with a 1-pixel low-pass filter. Scale bar, 1mm. **(b)** Confocal light microscopy of immunolabeled of teased fibers for CMTM6 (red) and MAG (green) validates adaxonal localization of CMTM6 in peripheral nerves of control and its absence in peripheral nerves of *Cmtm6*-cKo-mice at 2 mo. Scale bar, 5µm.

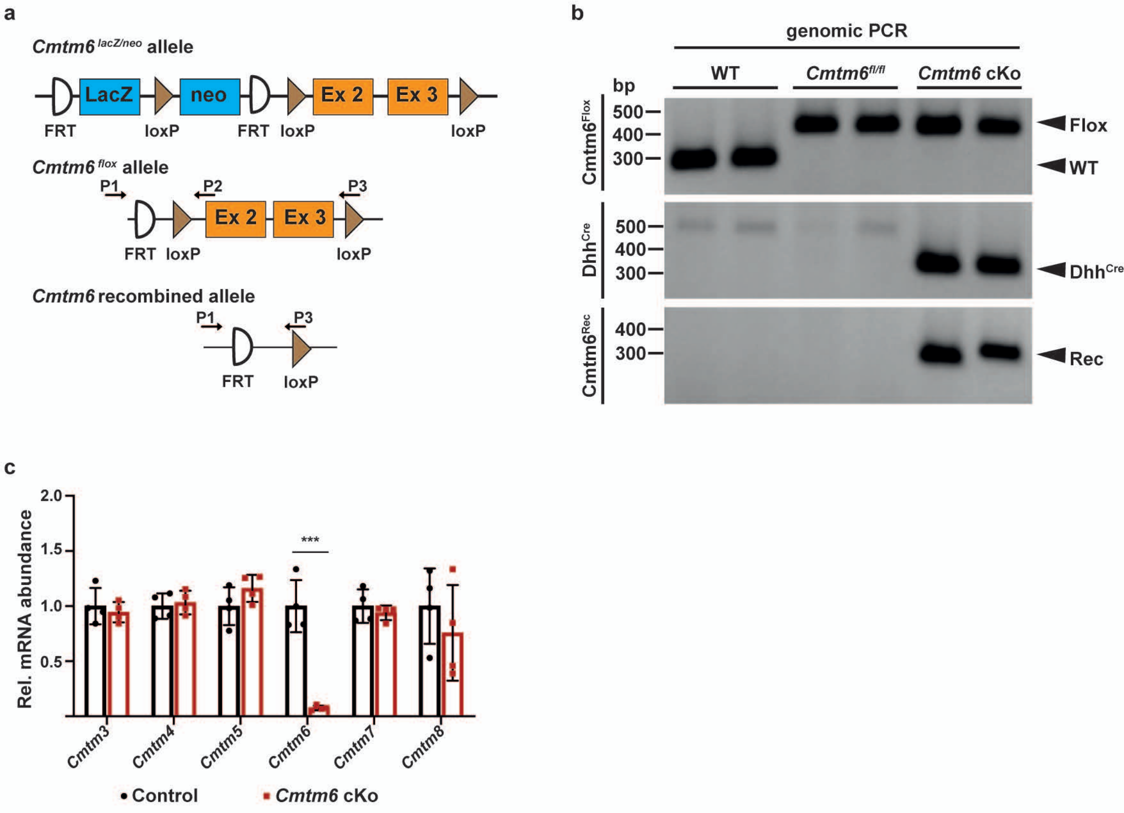

Supplementary Fig. 4. Conditional inactivation of the *Cmtm6* gene in Schwann cells. **(a)** Scheme of the engineered *Cmtm6* allele before and after recombination. Exons 2 and 3 of the *Cmtm6*^{flox} allele are flanked by loxP-sites for Cre-mediated recombination. Positions of PCR primers (P1, P2, P3) are indicated. **(b)** Genotyping PCR of DNA isolated from mouse ear punch biopsies identifies the indicated alleles. Upper image shows PCR product identifying wild-type (WT) and *Cmtm6*^{flox} alleles. Middle image shows PCR product identifying DhhCre mice. Lower image shows PCR product identifying recombined *Cmtm6* allele. Gel shows n=2 mice per genotype. **(c)** Transcript levels of mRNAs encoding members of the CMTM family in sciatic nerves dissected from *Cmtm6*-cKo and control at 2 mo. Note that *Cmtm6*-mRNA is virtually undetectable in *Cmtm6*-cKo nerves. Transcript levels were determined by qRT-PCR. n=4 per genotype; Two-way analysis of variance (ANOVA) with Sidak`s multiple comparisons test (*Cmtm3* P=0.9993; *Cmtm4* P=0.9998; *Cmtm5* P=0.8483; *Cmtm6* P=0.000001; *Cmtm7* P=0.9989; *Cmtm8* P=0.4664). For immunoblot and immunohistochemistry validating absence of CMTM6 expression *Cmtm6*-cKo nerves see Fig. 1i and Supplementary Fig. 3. Data are presented as mean +/- SD. n.s.=non-significant P>0.05, ***P<0.001. Source data are provided as a Source Data file.

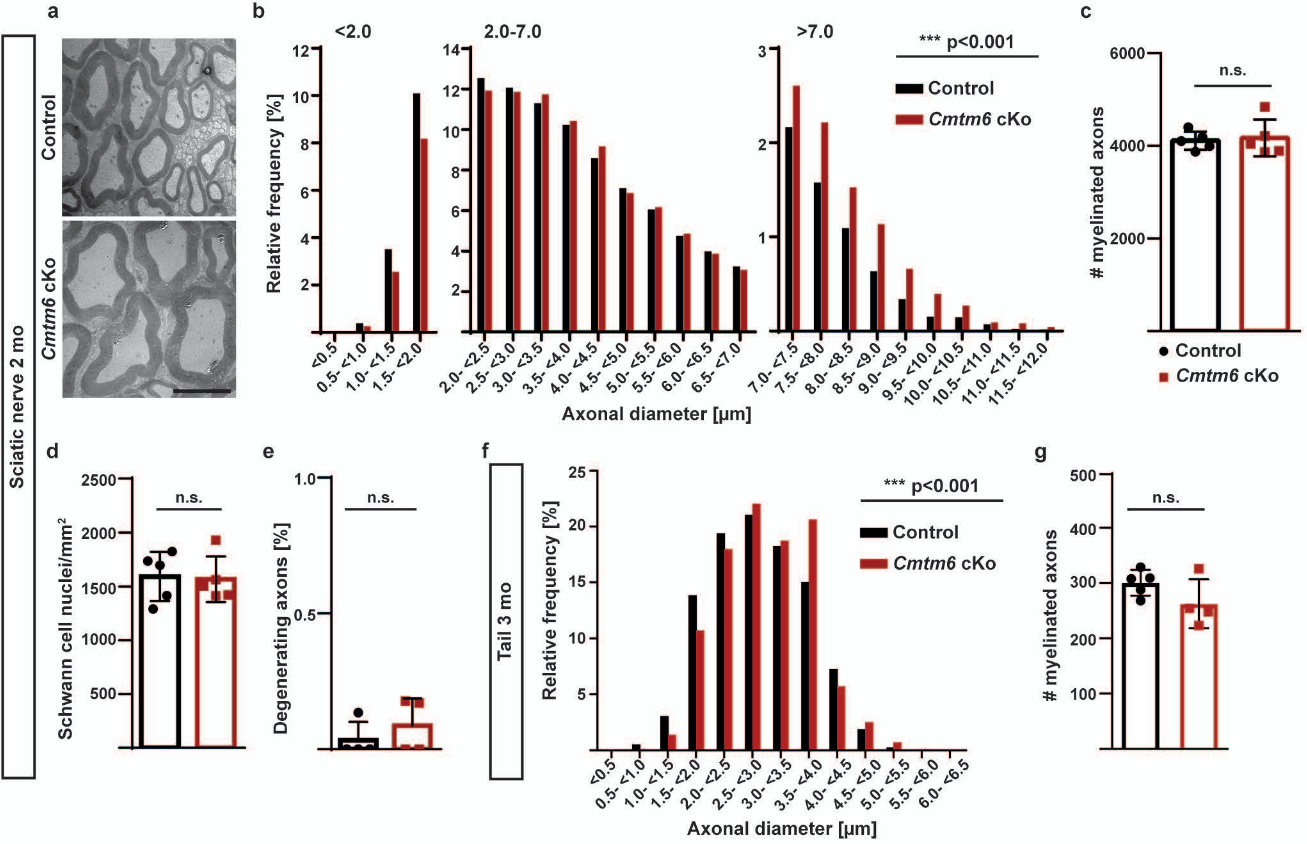

Supplementary Fig. 5. Axonal diameters are increased in sciatic nerves and caudal tail nerves of adult *Cmtm6*-cKo mice without affecting axonal survival.

(a) Representative electron micrographs of cross-sectioned sciatic nerves show increased axonal diameters in *Cmtm6*-cKo compared to control mice at 2 mo. Scale bar 5µm. **(b)** Genotype-dependent quantification of the diameters of myelinated axons on semi-thin sections confirms shift towards larger axonal diameters in sciatic nerves of *Cmtm6*-cKO-mice. Data are presented as frequency distribution with 0.5 µm bin width, n=19250 axons from n=5 control mice and n=18966 axons from n=5 *Cmtm6*-cKO mice; Mean axonal diameter $_{(\text{control+/-}\textit{Cmtm6}\text{-cKo})}$ = 3.16 µm+0.09 µm; P=1.216e^{-5} by two-sided Kolmogorow-Smirnow test of frequency distributions. **(c)** Quantitative assessment of semi-thin sections reveals normal numbers of myelinated axons in the sciatic nerve of *Cmtm6*-cKo-mice. n=5 mice per genotype; P=0.7781 by Two-tailed Student`s *t*-test. **(d)** Quantitative assessment of semi-thin sections reveals normal numbers of Schwann cell nuclei in the sciatic nerves of *Cmtm6*-cKo-mice. n=5 mice per genotype; P=0.8557 by Two-tailed Student`s *t*-test. **(e)** Genotype-dependent assessment of electron micrographs of cross-sectioned sciatic nerves reveals normal number of degenerating-appearing axonal profiles in *Cmtm6*-cKo-mice. n=4 per genotype; P=0.4132 by Two-tailed Student`s *t*-test. **(f)** Genotype-dependent quantification of the diameters of myelinated axons on semi-thin sections of cross-sectioned tails shows shift towards larger axonal diameters in 3-month old *Cmtm6*-cKO-mice. Data are presented as frequency distribution with 0.5 µm bin width, n=1627 axons from n=5 control mice and n=1198 axons from n=4 *Cmtm6*-cKO mice; Mean axonal diameter $_{(\text{control+/-}\textit{Cmtm6}\text{-cKo})}$ = 2.84 µm+0.14 µm; P=0.00012 by two-sided Kolmogorow-Smirnow test of frequency distributions. **(g)** Quantitative assessment on semi-thin sections reveals normal numbers of myelinated axons in the tails of *Cmtm6*-cKo-mice compared to control mice. n=5 control mice and n=4 *Cmtm6*-cKO mice, P=0.141 by Two-tailed Student`s *t*-test. Data in **c-e**, **g** are presented as mean +/- SD. n.s.=non-significant P>0.05, ***P<0.001. Source data are provided as a Source Data file.

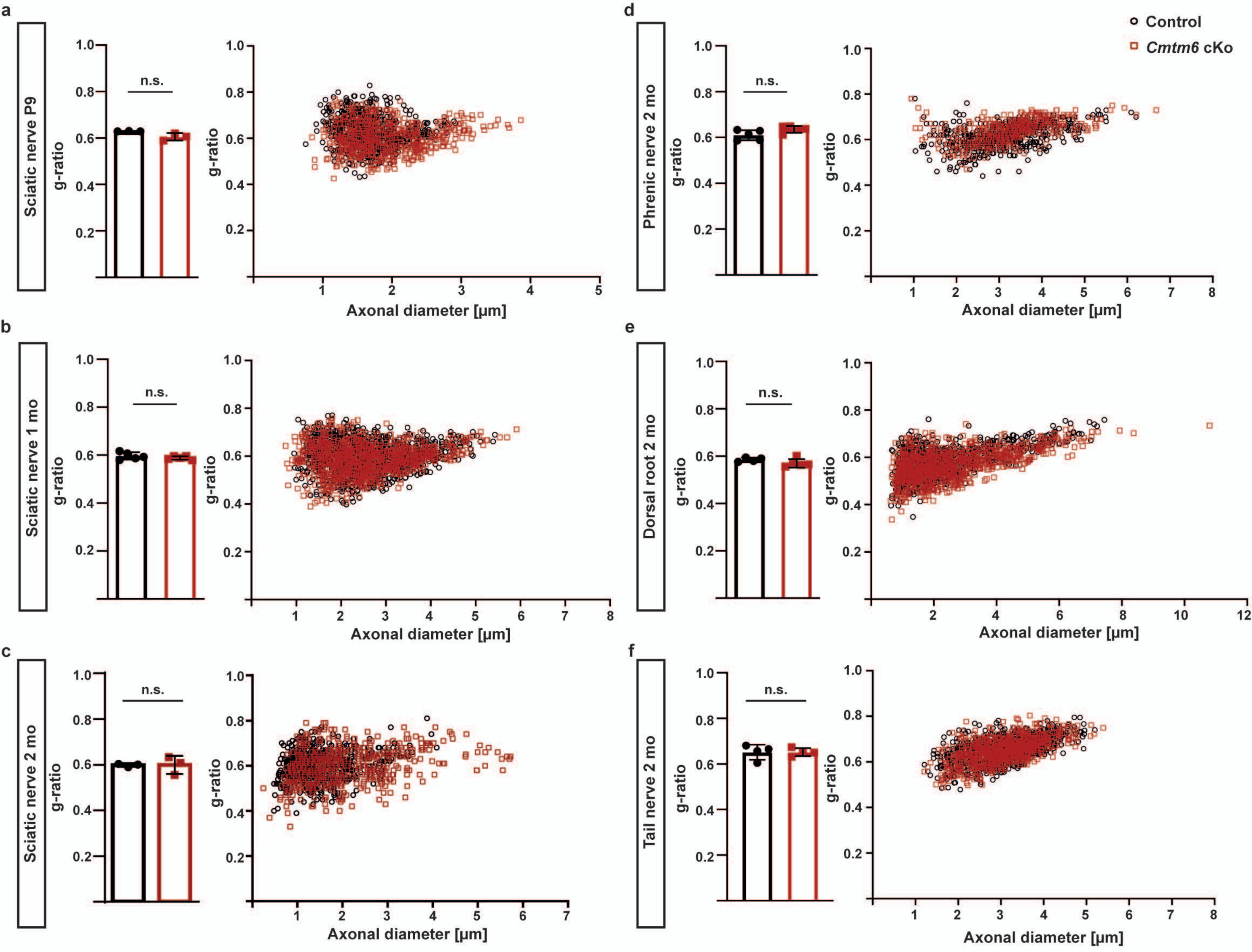

Supplementary Fig. 6. Appropriate myelin sheath thickness in peripheral nerves of *Cmtm6*-cKo mice.
(a) g-ratio analysis of electron micrographs of sciatic nerves cross sections shows appropriate myelin sheath thickness in *Cmtm6*-cKo-mice compared to control mice at P9. n=363-380 axons per mouse with n=3 mice per genotype; P=0.063 by Two-tailed Student`s *t*-test. **(b)** g-ratio analysis of electron micrographs of sciatic nerves cross sections shows appropriate myelin sheath thickness in *Cmtm6*-cKo-mice compared to control mice at 1 mo. n=181-187 axons per mouse with n=5 mice per genotype; P=0.2799 by Two-tailed Student`s *t*-test. **(c)** g-ratio analysis of electron micrographs from sciatic nerve cross sections identifies appropriate myelin sheath thickness in *Cmtm6*-cKo-mice at 2 mo. n=180 axons per mouse with n=3 mice per genotype; P=0.9785 by Two-tailed Student`s *t*-test. **(d)** g-ratio analysis of electron micrographs reveals appropriate myelin sheath thickness in *Cmtm6*-cKO phrenic nerves at 2 mo. n=505 axons from n=5 control mice; n=458 axons from n=5 *Cmtm6*-cKO mice; P=0.555 by Two-tailed Student`s *t*-test. **(e)** g-ratio analysis of electron micrographs from dorsal root cross sections shows appropriate myelin sheath thickness in *Cmtm6*-cKo-mice. n=166-188 axons per mouse with n=4 control and n=5 *Cmtm6*-cKo-mice; P=0.154 by Two-tailed Student`s *t*-test. **(f)** g-ratio analysis of semi-thin sections from tail nerve cross sections shows appropriate myelin sheath thickness in *Cmtm6*-cKo-mice. n=190 axons per mouse with n=4 mice per genotype; P=0.993 by Two-tailed Student`s *t*-test. Data are presented as mean +/- SD. n.s.=non-significant P>0.05. Source data are provided as a Source Data file.

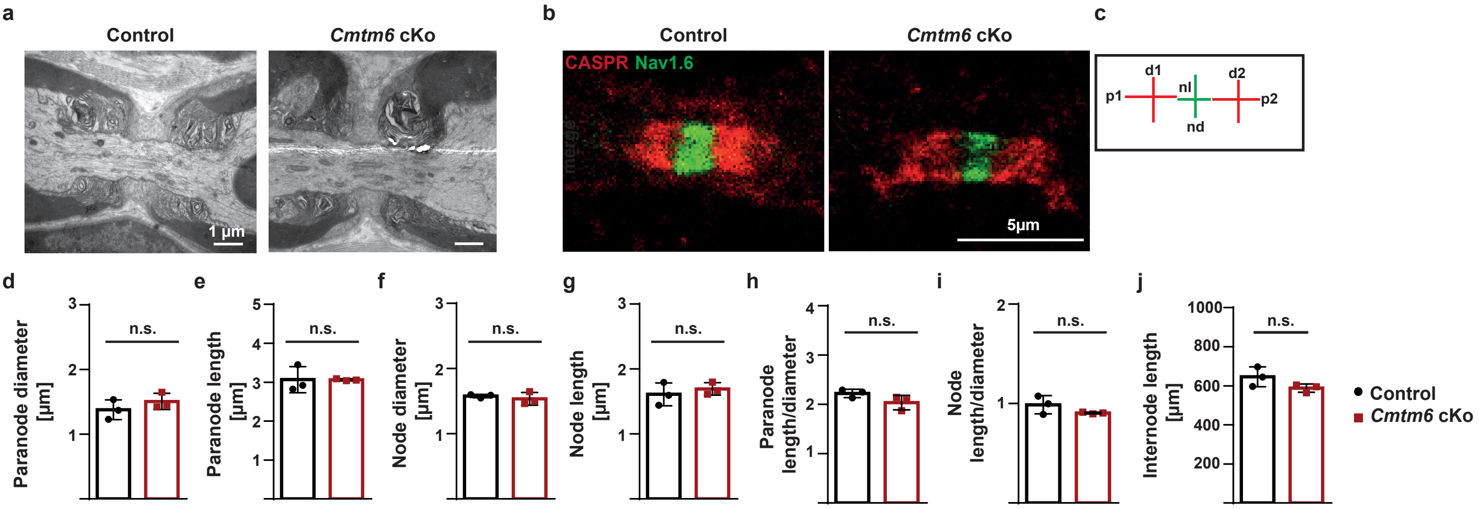

Fig. S7. Loss of CMTM6 does not affect nodal and paranodal dimensions or internode length.
(a) Electron micrographs of the nodes of Ranvier in longitudinally sectioned sciatic nerves of control and *Cmtm6* ckO mice at 2 mo. Scale bar, 1µm. **(b)** Immunolabeling of markers for the nodes of Ranvier (sodium channel Nav1.6, green) and paranodes (CASPR, red) on sciatic nerve teased fiber preparations dissected from *Cmtm6*-cKo and control mice at 2 mo. Scale bar, 5µm. **(c)** Scheme shows the parameters measured in **d** to **i**. p1=paranode length 1; p2= paranode length 2; d=diameter; nd=node diameter; nl=node length. **(d to e)** Genotype-dependent quantification of paranode diameter **(d)** and length **(e)** shows normal paranodal dimensions in *Cmtm6*-cKo-mice. Mean +/- SD, n>130 paranodes per mouse with n=3 mice per genotype; **d** P=0.3173 **e** P=0.9809; by Two-tailed Student`s *t*-test. n.s.=non-significant. **(f to g)** Genotype-dependent quantification of node diameter **(f)** and length **(g)** shows normal nodal dimensions in *Cmtm6*-cKo mice. Mean +/- SD, n>130 nodes per mouse with n=3 mice per genotype; **f** P=0.5089 **g** P=0.4868; by Two-tailed Student`s *t*-test. n.s.=non-significant. **(h to i)** Ratio of paranode length to diameter **(h)** or node length to diameter **(i)** shows normal paranodal and nodal dimension in *Cmtm6*-cKo-mice. Mean +/- SD, n>130 paranodes/nodes per mouse with n=3 mice per genotype; **h** P=0.1251 **i** P=0.1999; by Two-tailed Student`s *t*-test. n.s.=non-significant. **(j)** Genotype-dependent quantification on sciatic nerve teased fiber preparations reveals no difference in internode length between *Cmtm6*-cKo and control mice. Mean +/- SD; n=490 internodes from n=3 control mice and n=437 internodes from n=3 *Cmtm6*-cKo mice; P=0.1393 by Two-tailed Student`s *t*-test. n.s. = non-significant P>0.05,

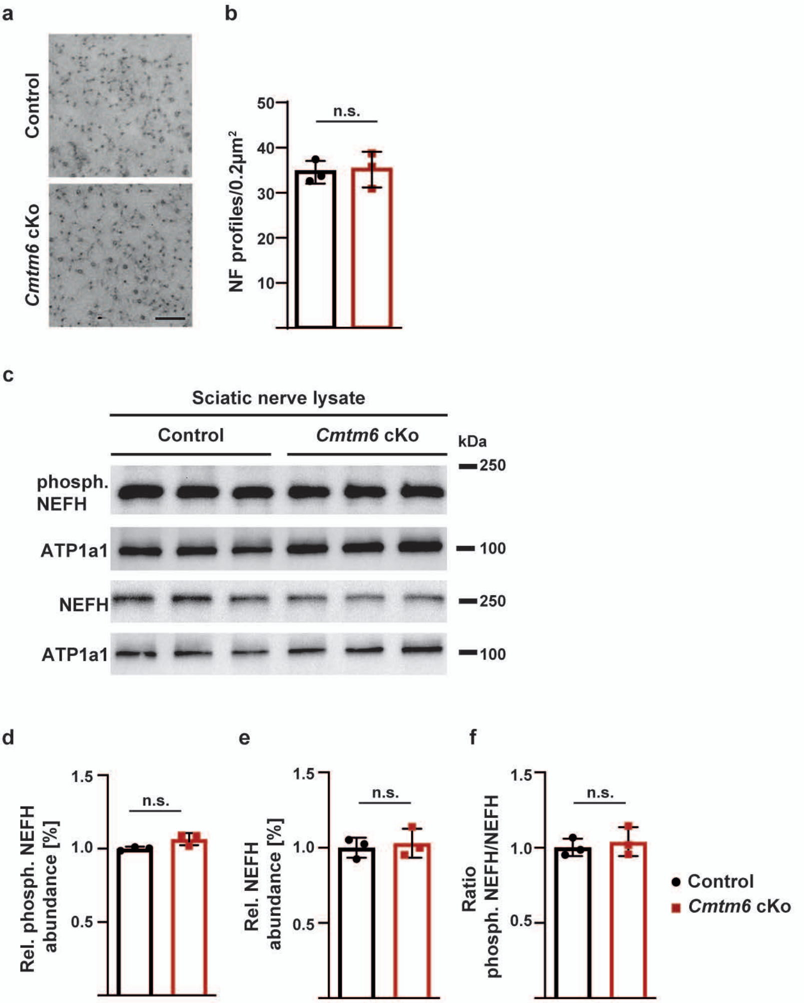

Supplementary Fig. 8. ***Cmtm6*-cKo nerves display normal neurofilament density and phosphorylation.**
(a) Representative electron micrographs of axonal neurofilaments in cross-sectioned sciatic nerves of *Cmtm6*-cKo and control mice. Scale bar, 200 nm. **(b)** Genotype-dependent quantification of the number of neurofilament profiles per 0.2µm2 on cross-sectioned sciatic nerves implies normal neurofilament density in *Cmtm6*-cKo compared to control mice (2 mo). Mean neurofilament number was assessed in n=30 axons per mouse with n=3 mice per genotype; P=0.8408 by Two-tailed Student`s *t*-test. **(c)** Immunoblot of sciatic nerve lysates of *Cmtm6*-cKo and control mice at 2 mo using antibodies directed against phosphorylated neurofilament-H (NEFH) or pan-NEFH as quantified in **d** to **f**. ATP1a1 serves as control. Blots showing n=3 mice per genotype. **(d to f)** Quantification of immunoblots in **c** implies normal phosphorylation of NEFH in *Cmtm6*-cKo compared to control mice. n=3 mice per genotype; **d** P=0.0603 **e** P=0.6835 **f** P= 0.5988, by Two-tailed Student`s *t*-test. Data in **b, d-f** are presented as mean +/- SD. n.s.=non-significant P>0.05. Source data are provided as a Source Data file.

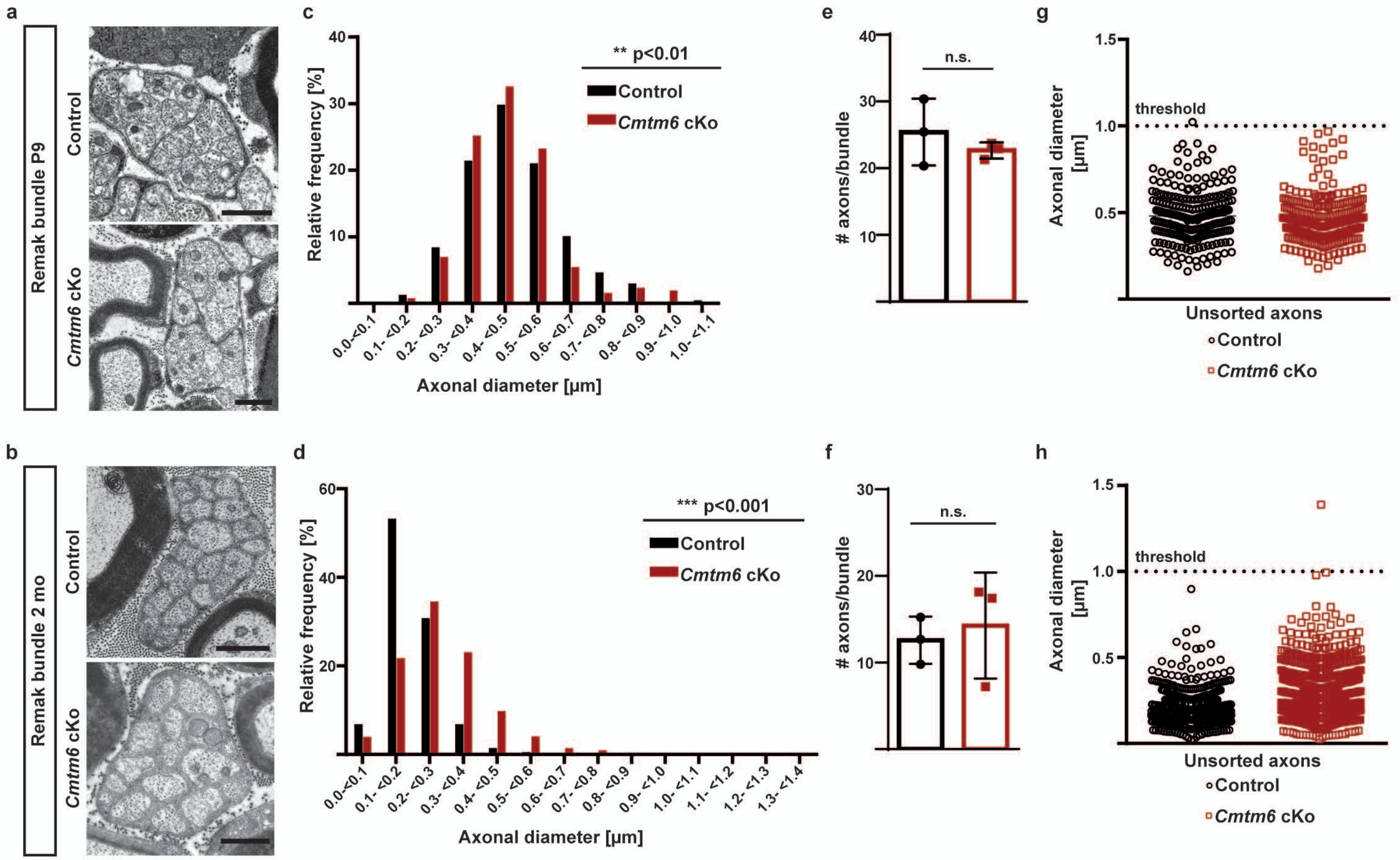

Supplementary Fig. 9. Abnormally increased diameters of non-myelinated, Remak-associated axons but normal radial sorting when CMTM6 is lacking from Schwann cells.

(a and **b)** Electron micrographs showing Remak bundles in *Cmtm6*-cKo and control sciatic nerves at P9 and 2 mo. Scale bar, 1µm. **(c** and **d)** Genotype-dependent quantification reveals increased diameters of non-myelinated, Remak-associated axons in *Cmtm6*-cKo compared to control mice at P9 and 2 mo. Data are presented as frequency distribution with 0.1 µm bin width; **c** n=690 axons from n=3 control mice and n=709 axons from n=3 *Cmtm6*-cKO mice; Mean axonal diameter $_{\text{(control+/-}\textit{Cmtm6}\text{-cKo)}}$ = 0.46 µm+0.02 µm; P=0.002 by two-sided Kolmogorow-Smirnow test of frequency distributions; **d** n=2522 axons from n=3 control mice and n=2377 axons from n=3 *Cmtm6*-cKO mice; Mean axonal diameter $_{\text{(control+/-}\textit{Cmtm6}\text{-cKo)}}$ = 0.19µm+0.08 µm; ***, P=2.2e^{-16} by two-sided Kolmogorow-Smirnow test of frequency distributions. Myelinated axons at P9 and 1 mo see Supplementary Fig. 10. **(e and f)** Genotype-dependent assessment shows unchanged number of non-myelinated, Remak-associated axons per bundle in *Cmtm6*-cKo-mice, implying normal radial sorting. n=3 mice per genotype; **e** P= 0.4057 **f** P= 0.683; by Two-tailed Student`s *t*-test. Pro-myelinated axons at P9 see Supplementary Fig. 10. **(g and h)** Genotype-dependent assessment shows normal threshold diameter (1 µm) of non-myelinated, Remak-associated axons in *Cmtm6*-cKo-mice, implying normal radial sorting. Data are presented as data clouds; **g** n=690 axons from 3 control mice and n=709 axons from 3 *Cmtm6*-cKo mice **h** n=2522 axons from 3 control mice and n=2377 axons from 3 *Cmtm6*-cKo mice. Data in **e-f** are presented as mean +/- SD. n.s.=non-significant P>0.05, **P<0.01, ***P<0.001. Source data are provided as a Source Data file.

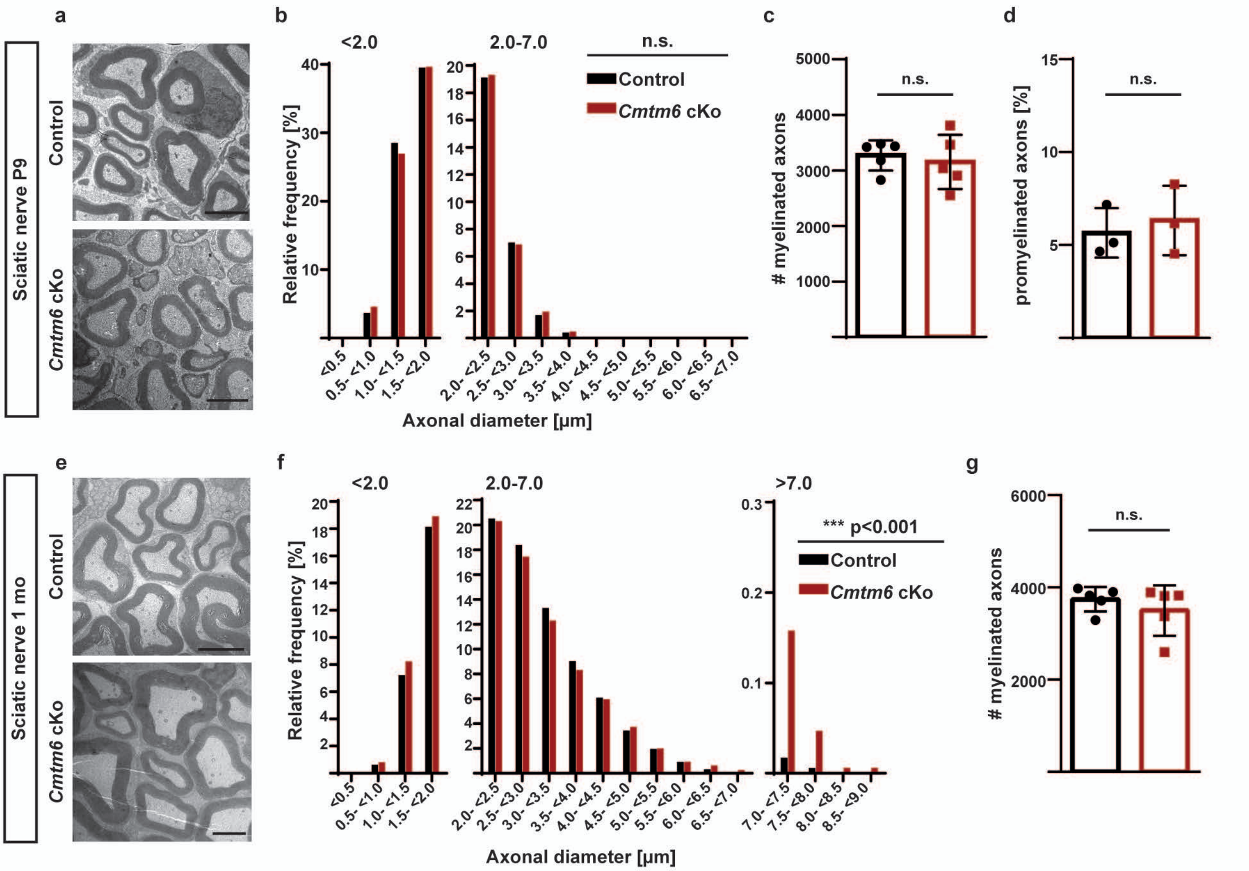

Supplementary Fig. 10. Developmental emergence of increased diameters of myelinated axons in *Cmtm6*-cKo mice.
(a) Electron micrographs of cross-sectioned sciatic nerves show no change of axonal diameters in *Cmtm6*-cKo-mice compared to control mice at P9. Scale bar, 2.5 µm. **(b)** Genotype-dependent quantification of the diameters of myelinated axons in sciatic nerves at P9 shows no significant difference between *Cmtm6*-cKo and control mice. Data are presented as frequency distribution with 0.5 µm bin width; n=16350 axons from n=5 control mice and n=15775 axons from n=5 *Cmtm6*-cKO mice; Mean axonal diameter$_{\text{(control+/-}\textit{Cmtm6}\text{-cKo)}}$ = 1.77 µm+0.005 µm; P=0.3648 by Two-tailed Student`s *t*-test. **(c, d)** Genotype-dependent quantification of the number of myelinated **(c)** and pro-myelinated **(d)** axons on semi-thin sections of sciatic nerves shows no change between *Cmtm6*-cKo and control mice. **c** n=5 mice per genotype, P=0.6579 **d** n=3 mice per genotype, P=0.6424; by Two-tailed Student`s *t*-test. **(e)** Electron micrographs of cross-sectioned sciatic nerves display increased axonal diameters in 1- month old *Cmtm6*-cKo-mice compared to control mice. Scale bar, 2.5 µm. **(f)** Genotype-dependent quantification of the diameters of myelinated axons in sciatic nerves at 1 mo reveals a shift towards increased axonal diameters in *Cmtm6*-cKo compared to control mice. Data are presented as frequency distribution with 0.5 µm bin width; n=17578 axons from n=5 control mice and n=16966 axons from n=5 *Cmtm6*-cKO mice; Mean axonal diameter$_{\text{(control+/-}\textit{Cmtm6}\text{-cKo)}}$ = 2.68 µm+0.12 µm; P=2.2e^{-16} by two-sided Kolmogorow-Smirnow test of frequency distributions. **(g)** The number of myelinated axons is unchanged in sciatic nerves of *Cmtm6*-cKo compared to control mice at 1 mo. n=5 mice per genotype, P=0.4036 by Two-tailed Student`s *t*-test. g-ratios see Supplementary Fig. 6. Data in **c,d,e** are presented as mean +/- SD. n.s.=non-significant P>0.05, ***P<0.001. Source data are provided as a Source Data file.

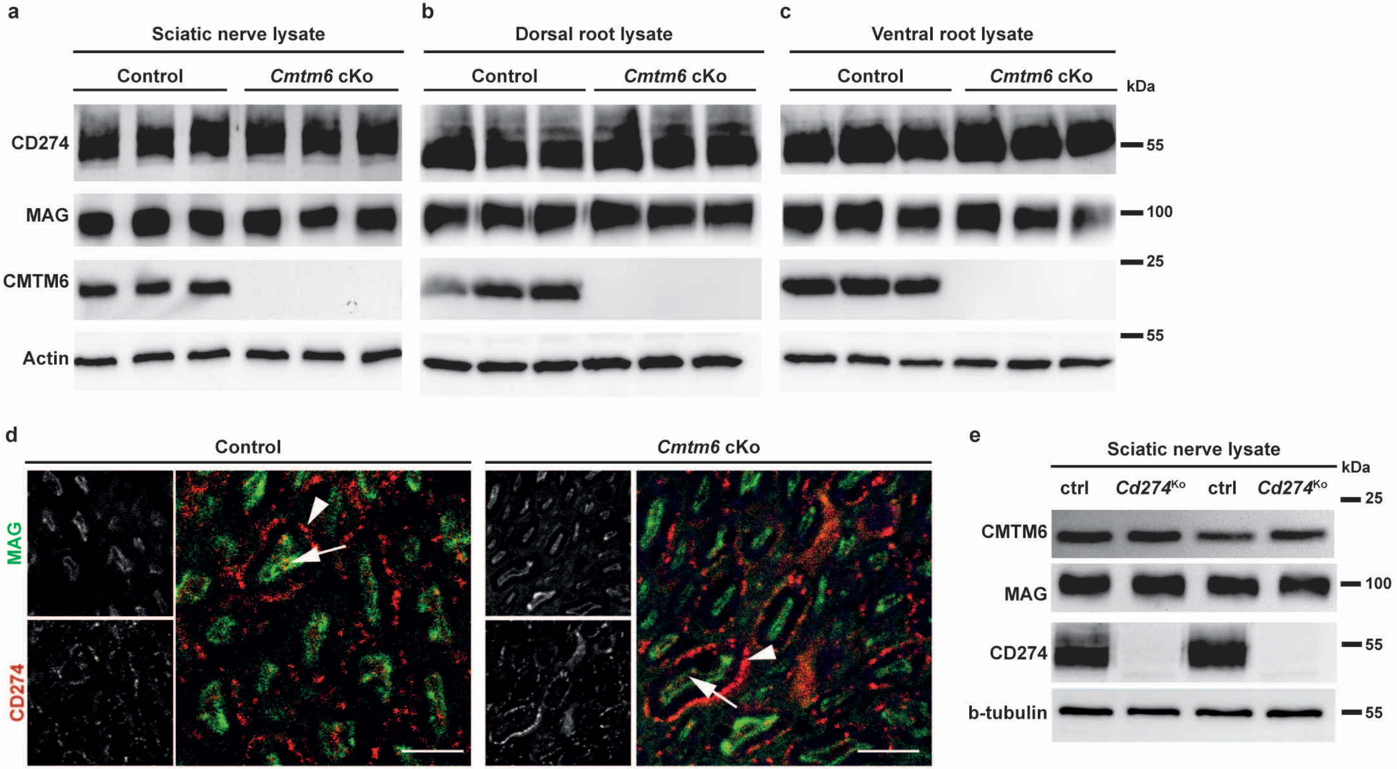

Supplementary Fig. 11. Abundance and localization of CD274 in the PNS is independent of CMTM6
(a-c) Immunoblot analysis of lysates of sciatic nerves **(a)**, dorsal roots **(b)** and ventral roots **(c)** dissected from *Cmtm6*-cKo and control mice at 2 mo indicates normal abundance of CD274 and MAG. CMTM6 was detected as genotype control; actin serves as control. Blots showing n=3 mice per genotype. **(d)** Immunolabeling of cross-sectioned sciatic nerves dissected from *Cmtm6*-cKo and control mice at 2 mo reveals no change in the preferentially abaxonal localization of CD274 (red; arrowheads). MAG (green; arrows) serves as marker preferentially labeling adaxonal myelin. Scale bars, 5 µm. **(e)** Immunoblotting of sciatic nerves lysates from adult *Cd274*-Ko and control mice shows unchanged abundance of CMTM6 and MAG when CD274 is lacking. Beta-tubulin serves as control. Blot showing n=2 mice per genotype. Source data are provided as a Source Data file.

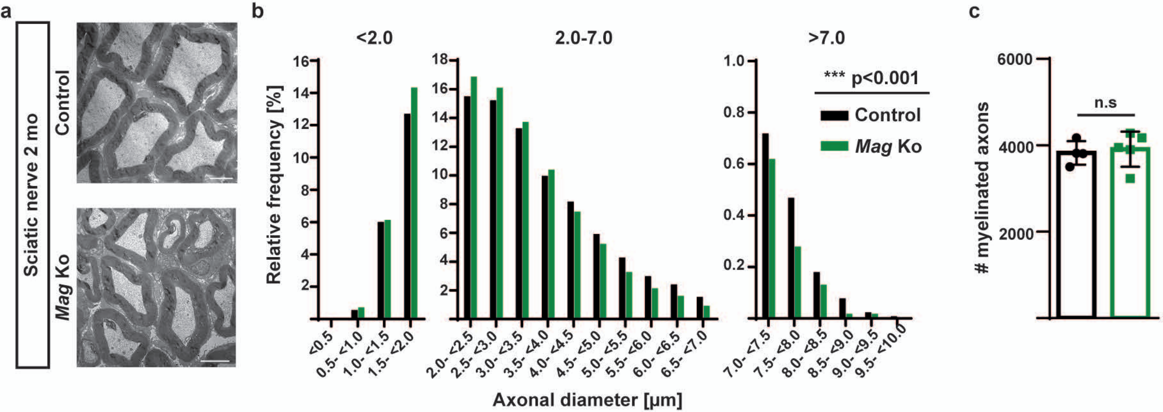

Supplementary Fig. 12. Axonal diameters are reduced in the absence of MAG

(a) Electron micrographs of cross-sectioned sciatic nerves show decreased axonal diameters in *Mag* Ko compared to control mice at P75. Scale bar 2.5 µm. **(b)** Genotype-dependent quantification confirms abnormally reduced diameters of myelinated axons in *Mag*-Ko compared to control sciatic nerves[15]. Data are presented as frequency distribution with 0.5 µm bin width; n=12793 axons from n=4 control mice and n=16790 axons from n=5 *Mag*-Ko mice; Mean axonal diameter $_{(\text{control}+/-\textit{Mag}\text{-Ko})}$ = 3.26µm-0.17µm; P<2.2e^{-16} by two-sided Kolmogorow-Smirnow test of frequency distributions. **(c)** Quantitative assessment shows unchanged numbers of myelinated axons in Mag-Ko sciatic nerves. Data are presented as mean +/- SD, n=4 control and n=5 Mag-Ko mice; P=0.7413 by Two-tailed Student`s t-test. n.s.=non-significant P>0.05, ***P<0.001. Source data are provided as a Source Data file.
