# Supplementary material for: CMTM6 expressed on the adaxonal Schwann cell surface restricts axonal diameters in peripheral nerves
Source: Nat Commun. 2020 Sep 9;11:4514. doi: 10.1038/s41467-020-18172-7 (PMC7481192; doi:10.1038/s41467-020-18172-7)
Supplement: Supplementary file 2 — Description of Additional Supplementary Files [file 41467_2020_18172_MOESM2_ESM.pdf]

Description of Additional Supplementary Files

Title: Supplementary Data 1.

Description: Proteomic analysis of axogliasome-enriched fractions. Biochemically isolated axogliasome-enriched fractions were subjected to proteolytic digestion using filter-aided sample preparation. Tryptic peptides (corresponding to 200 ng protein) were analysed by LC-IMS-MS on a Synapt-G2S instrument using 90 min gradients. Data were processed with ProteinLynx Global SERVER (PLGS, version 3.02) and the software tool ISOQuant. Proteins (FDR 1%; 2 peptides/protein) and peptides (FDR < 1%; ≥6 amino acids) were identified searching against the UniProt mouse reference proteome (UniProtKB release 2013_01, 16,580 entries). In the present table identified proteins along with their calculated relative amounts [ppm: parts per million (w/w) of total protein]. To be included in the final list peptides had to be identified in at least three biological replicates (two peptides/protein). Moreover, only peptides with a PLGS identification score >= 5.5 were considered.
