# Supplementary material for: CMTM6 expressed on the adaxonal Schwann cell surface restricts axonal diameters in peripheral nerves
Source: Nat Commun. 2020 Sep 9;11:4514. doi: 10.1038/s41467-020-18172-7 (PMC7481192; doi:10.1038/s41467-020-18172-7)
Supplement: Supplementary file 4 — Reporting Summary [file 41467_2020_18172_MOESM4_ESM.pdf]

nature research

Corresponding author(s): Dr. Hauke B. Werner

Last updated by author(s): 2020_07_23

Reporting Summary

Nature Research wishes to improve the reproducibility of the work that we publish. This form provides structure for consistency and transparency in reporting. For further information on Nature Research policies, see Authors & Referees and the Editorial Policy Checklist .

Statistics

For all statistical analyses, confirm that the following items are present in the figure legend, table legend, main text, or Methods section.

n/a	Confirmed	
[ ]	[x]	The exact sample size (*n*) for each experimental group/condition, given as a discrete number and unit of measurement
[ ]	[x]	A statement on whether measurements were taken from distinct samples or whether the same sample was measured repeatedly
[ ]	[x]	The statistical test(s) used AND whether they are one- or two-sided<br>*Only common tests should be described solely by name; describe more complex techniques in the Methods section.*
[ ]	[ ]	A description of all covariates tested
[ ]	[x]	A description of any assumptions or corrections, such as tests of normality and adjustment for multiple comparisons
[ ]	[x]	A full description of the statistical parameters including central tendency (e.g. means) or other basic estimates (e.g. regression coefficient) AND variation (e.g. standard deviation) or associated estimates of uncertainty (e.g. confidence intervals)
[ ]	[x]	For null hypothesis testing, the test statistic (e.g. *F*, *t*, *r*) with confidence intervals, effect sizes, degrees of freedom and *P* value noted<br>*Give P values as exact values whenever suitable.*
[x]	[ ]	For Bayesian analysis, information on the choice of priors and Markov chain Monte Carlo settings
[x]	[ ]	For hierarchical and complex designs, identification of the appropriate level for tests and full reporting of outcomes
[x]	[ ]	Estimates of effect sizes (e.g. Cohen's *d*, Pearson's *r*), indicating how they were calculated

Our web collection on statistics for biologists contains articles on many of the points above.

Software and code

Policy information about availability of computer code

Data collection	Proteomics: ProteinLynx Glocal SERVER (PLGS); Version 3.02<br>post-processing of Proteomics: ISOQuant; Version 1.8<br>https://www.uniprot.org/statistics/Swiss-Prot<br>Plethysmography:LabChart-software (ADInstruments); Version 8.1.13

Data analysis

Microscopy and data analysis:
Zeiss Zen2011 and ZEN 2012 blue edition
Zeiss; LAS X software, Leica-microsystems
ImageJ software (https://imagej.nih.gov/ij/); Version 1.51s and 1.50b
ImSpector software package (Max-Planck Innovation); Version 14.1-16.1
LAS AF lite, Version 2.6.0.
Matlab R2015b

Statistics and data representation:
GraphPad Prism (GraphPad Software, Inc., San Diego, United States;https://www.graphpad.com); Version 5.0 and 8.0
RStudio (https://www.rstudio.com/); Version 3.4.1; for Komolgorov-Smirnov test for frequency distributions: custom code using R-Studio 4.3.1 (further information see method section on statistics, written by Drini Morina); R code:https://github.com/MariaEichel/FrequencyDistributions.git
Excel 2013
bioRender; http://biorender.com

For manuscripts utilizing custom algorithms or software that are central to the research but not yet described in published literature, software must be made available to editors/reviewers. We strongly encourage code deposition in a community repository (e.g. GitHub). See the Nature Research guidelines for submitting code & software for further information.

Data

Policy information about availability of data

All manuscripts must include a data availability statement. This statement should provide the following information, where applicable:

- Accession codes, unique identifiers, or web links for publicly available datasets
- A list of figures that have associated raw data
- A description of any restrictions on data availability

All data generated or analysed during this study are included in this published article (and its supplementary information files and Sourca Data file). The source data as well as original immunoblot scans underlying Figures 1-5 and Supplementary Figure 4-12 are provided as a Source Data File. The mass spectrometry proteomics data are provided as Supplementary Table 1. STED microscopy data underlying Figure 1 and Supplementary Figure 3 are available from the corresponding author on reasonable request.

Field-specific reporting

Please select the one below that is the best fit for your research. If you are not sure, read the appropriate sections before making your selection.

☒ Life sciences ☐ Behavioural & social sciences ☐ Ecological, evolutionary & environmental sciences

For a reference copy of the document with all sections, see nature.com/documents/nr-reporting-summary-flat.pdf

Life sciences study design

All studies must disclose on these points even when the disclosure is negative.

Sample size

Number of used animals is as small as reasonably possible to obtain statistically significant results. For animal experiments accepted group sizes are used as it is common in the field (see Patzig, J. et al. Septin/anillin filaments scaffold central nervous system myelin to accelerate nerve conduction. Elife 5, e17119 (2016); Patzig, J. et al. Proteolipid protein modulates preservation of peripheral axons and premature death when myelin protein zero is lacking. Glia 64, 155–174 (2016); Fledrich, R. et al. Soluble neuregulin-1 modulates disease pathogenesis in rodent models of Charcot-Marie-Tooth disease 1A. Nat. Med. (2014). doi:10.1038/nm.3664; Fledrich, R. et al. NRG1 type I dependent autoparacrine stimulation of Schwann cells in onion bulbs of peripheral neuropathies. Nat. Commun. 10, 1467 (2019); Fledrich, R. et al. A rat model of Charcot–Marie–Tooth disease 1A recapitulates disease variability and supplies biomarkers of axonal loss in patients. Brain 135, 72–87 (2012))
No statistical method was used to predetermine sample size.

Data exclusions

Outlier test by GraphPad, http://graphpad.com/quickcalcs/Grubbs1.cfm as described in methods section on quantifications and statistical analysis. Except for Nerve Conduction velocity meassurements (Fig. 3) no outliers were identified, and thus no datapoints were excluded.

Replication

In general: Replication in vivo was performed by biological replicates of at least n=3.

In more detail:
To test for accuracy of semi-automated quantification method of axonal diameter analysis one round of testing was performed with one full n=5 dataset by two separate persons. Analyzed data did not vary between observers and results confirmed what was previously seen on electron microscopic level (data not shown). Hence, the semi-automated method was applied for evaluating axonal diameters for all datasets with 4 or 5 biological replicates per genotype (further detail see method section and figure legends).
Western Blots and IHC staining: were replicated 3 times with at least n=3 biological replicates. For immunoblots (Figure 1b, f; Supplementary Figure 11e) representative images of 3 - 5 independent experiments are shown.
For qRT PCR 4 biological replicates with 4 technical replicates each were used.
Representative micrographs of electron microscopic analysis were chosen independently from the obtained images of the 3-5 different biological replicates per genotype. Electron micrograph of immunogold-labeling in Fig. 1g was chosen from a collection of 20 images from 2

independent experiments showing identical outcome.
STED image (Fig1) represents the results of 3 independent experiments. STED image (Supplementary Fig. 3a) represents the results of 20 lines profiles derived from 7 independent fields of view of one experiment.
For fluorescent labeling, lacZ staining and/or confocal microscopy representative images were chosen from 3 independent experiments showing identical outcome.

All attempts at replication were successful. Number of independent replicates for each experiments and replication for representative images are indicated either in the respective figure legend and in the method section under "Statistics and Reproducibility".

Randomization

For morphological and IHC analyses random images were taken and analyzed blinded to the genotype as stated in the method section. For animal studies, organisms were allocated randomly to the experimental groups with only considering the determined genotypes.

Blinding

Investigators were blinded to group allocation during data collection and analysis (behavior, electrophysiology, histology). Selection of animal samples out of different experimental groups for molecular biology/histology/biochemistry was performed randomly and in a blinded fashion. For immunoblotting experiments (step: loading gels) investigator was not blinded to genotypes anymore to facilitate accurate loading.

Reporting for specific materials, systems and methods

We require information from authors about some types of materials, experimental systems and methods used in many studies. Here, indicate whether each material, system or method listed is relevant to your study. If you are not sure if a list item applies to your research, read the appropriate section before selecting a response.

Materials & experimental systems

n/a	Involved in the study
☐	☒ Antibodies
☒	☐ Eukaryotic cell lines
☒	☐ Palaeontology
☐	☒ Animals and other organisms
☒	☐ Human research participants
☒	☐ Clinical data

Methods

n/a	Involved in the study
☒	☐ ChIP-seq
☒	☐ Flow cytometry
☒	☐ MRI-based neuroimaging

Antibodies

Antibodies used

Primary Antibodies:
Actin Chemicon Cat# MAB1501; 1:2000
alpha-Tubulin Sigma Cat# SAB2102603; 1:5000
ATPaseα1 Abcam Cat# ab7671; 1:2500
betaII spectrin BD Biosciences Cat# 612563
CASPR Neuromabs Cat# clone K65/35; 1:500
CD274/PDL1 Abcam Cat# ab213480; 1:500 (IB/IHC)
CNP Sigma Cat# C5922; 1:1000
CMTM6 OriGene Cat# TA322304; 1:500 (IB);1:200 (IHC); 1:100 (IEM)
MAG (clone 513) Chemicon, Cat# MB1567; 1:500 (IB); 1:50 (IHC)
MPZ J. Archelos-Garcia; 1:2000
Nav1.6 Almonelabs Cat# ASC-009; 1:500
NFASC155 Prof. Peter Brophy; 1:1000
PMP2 PTG Cat# 12717-1-AP; 1:1000
SMI31 Covance Cat #SMI31P; 1:500
SMI32 (NEFH) Covance Cat# SMI32-P; 1:500

Secondary Antibodies:
Sheep anti-mouse Dianova Cat# 515-005-003; 1:100
Goat-anti rabbit Dianova Cat# 111-005-003; 1:100
STAR580 Abberior Cat# ST580-0002; 1:100
STAR635P Abberior Cat# ST635P-002; 1:100
HRP-goat anti-mouse IgG Dianova Cat# 115-03-003; 1:10000
HRP-goat anti-rabbit IgG Dianova Cat# 111-035-003; 1:10000
donkey α-mouse-Alexa488 Invitrogen Cat# A21206; 1:1000
donkey α-rabbit-Alexa488 Invitrogen Cat# A21206; 1:1000
donkey α-mouse-Alexa555 Invitrogen Cat# A21202; 1:1000
donkey α-rabbit-Alexa555 Invitrogen Cat# A21202; 1:1000

Validation

CMTM6 antibody (OriGene) was successfully validated using conditional knock-out mice lacking CMTM6 (Fig 1 i, j) by immuno

blot and IHC. CD274/PDL-1 antibody was successfully validated using knock-out mice lacking CD274 (Supplementary Fig. 11). MAG antibody was succesfully validated using knock.out mice lacking MAG (Figure 5). All other antibodies are commonly used in the field and were already published and used according to manufactures websites or publication and have been used extensively in our laboratory (e.g. Patzig, J. et al. Septin/anillin filaments scaffold central nervous system myelin to accelerate nerve conduction. Elife 5, e17119 (2016); Patzig, J. et al. Proteolipid protein modulates preservation of peripheral axons and premature death when myelin protein zero is lacking. Glia 64, 155–174 (2016); Erwig, M. S. et al. Anillin facilitates septin assembly to prevent pathological outfoldings of central nervous system myelin. Elife 8, (2019); Siems et al. Proteome profile of peripheral myelin in healthy mice and in a neuropathy model, eLife 2020;9:e51406)

More information about dilution and manufacturer given in the respective method section and the reporting summary. Validation information from manufacturers Website below.

Actin Chemicon Cat# MAB1501; - Routinely evaluated by the company via Western Blot on A431 lysates.

alpha-Tubulin Sigma Cat# SAB2102603; validated by the company in placenta tissue lysate and mouse brain lysate (IB); in Mouse brain stem (IHC)

ATPaseα1 Abcam Cat# ab7671; validated by 233 citations (e.g. Vangeel et al 2020; Internation Journal of Molecular Sciences)
betaII spectrin BD Biosciences Cat# 612563; validated by 16 citations (e.g. Dubey et al 2020 eLife; Costa, A.R. 2020, eLife; DÈste et al 2017, Proceedings of the National Academy of Sciences of the United States of America)

CASPR Neuromabs Cat# clone K65/35; validated by the company on rat brain membrane tissue lysate (IB)

CD274/PDL1 Abcam Cat# ab213480; validated by the company via on RAW264.7 cell line and mouse placenta lysate (IB/IHC) and during analysis performed in this paper using Cd274 Ko sciatic nerve lysate (IB)

CNP Sigma Cat# C5922; validated by the company using fresh bovine whole brain extract (IB)

CMTM6 OriGene Cat# TA322304; 1:500 (IB);see above

MAG (clone 513) Chemicon, Cat# MB1567; see above

MPZ J. Archelos-Garcia; validated by Archelos, J. J. et al. Production and characterization of monoclonal antibodies to the extracellular domain of P0. J. Neurosci. Res. 35, 46–53 (1993).

Nav1.6 Almonelabs Cat# ASC-009; validated by the company using rat brain (IB), mouse hippocampus slices or rat DRG primary culture (IHC) and by several oublications (e.g. Hernandes-Plata E., et al. 2012 Int.J. cancer)

NFASC155 Prof. Peter Brophy; validated by Tait, S. et al. An Oligodendrocyte Cell Adhesion Molecule at the Site of Assembly of the Paranodal Axo-Glial Junction. J. Cell Biol. 150, 657–666 (2000)

PMP2 PTG Cat# 12717-1-AP; validated by the company using pig spinal chord tissue, human brain tissue and mouse testis tissue (IB) or human glioma tissue (IHC)

SMI31 Covance Cat #SMI31P; validted by >5 publication (e.g.Raina AK, Takeda A, Nunomura A,Perry G, Smith MA. Genetic evidence for oxidative stress in Alzheimer's disease. Neuroreport 10:1, 1999).

SMI32 (NEFH) Covance Cat# SMI32-P; validated by the company (now called bioLegend) on mouse brain tissue, mouse thalamus tissue and rat cerebellum (IHC) and via IB on human, rat and murine brain lysate and by > 5 publication (e.g. Trapp BD, Peterson J, Ransohoff RM, Rudick R, Mörk S, Bö L. Axonal transection in the lesions of multiple sclerosis. N Eng J Med 338:278-85, 1998.).

Animals and other organisms

Policy information about studies involving animals; ARRIVE guidelines recommended for reporting animal research

Laboratory animals

Magnull mice; also termed Mag Ko (Montag, D. et al. Mice deficient for the glycoprotein show subtle abnormalities in myelin. Neuron 13, 229–246 (1994))
Cd274null mice; also termed Cd274 Ko (Dong, H. et al. B7-H1 Determines Accumulation and Deletion of Intrahepatic CD8+ T Lymphocytes. Immunity 20, 327–336 (2004))
PlpCreERT2 mice: Leone, D. P. et al. Tamoxifen-inducible glia-specific Cre mice for somatic mutagenesis in oligodendrocytes and Schwann cells. Mol. Cell. Neurosci. 22, 430–440 (2003)
Dhh-Cre mice: Jaegle, M. et al. The POU proteins Brn-2 and Oct-6 share important functions in Schwann cell development. Genes Dev. 17, 1380–1391 (2003)
Cmtm6tm1c(EUCOMM)Wtsi termed Cmtm6flox mice; upon crossbreeding with Dhh-Cre or PlpCreERT2 the following mouse strains were yielded: Cmtm6flox/flox;DhhCre (Cmtm6 cKo mice) and Cmtm6flox/flox; PlpCreERT2 (Cmtm6 iKo mice); Crossbreeding Cmtm6 cKo with Mag Ko mice resulted in double knock-out mice termed Cmtm6flox/flox;DhhCre;Magnull mice

Mice were housed in mouse facility of the Max Planck Institute of Experimental Medicine with controlled ventilation (inward airflow, exhaust to the outside), temperature (21+/-2°C) and controlled humidity (80%) in individually ventilated cages. Mice

were group-housed 3-5 mice per cage in a 12-hour dark/light cycle with ad libitum access to food and water. Experimental mice were male unless indicated otherwise and were analyzed together with littermate controls as far as possible. For tamoxifen-injection both control (Cmtm6flox/flox) and Cmtm6 iKo (Cmtm6flox/flox; PlpCreERT2) were injected with tamoxifen. Exact age of mice is indicated in the figure legends. Briefly mice were analysed between P9 and P75 as given in the Figure legends except Cmtm6 iKo and respective control mice which were analysed at the age of 4 and 8 months as given in the Figure legend.

Wild animals	Study did not involve wild animals
Field-collected samples	Study did not involve field-collected samples
Ethics oversight	All animal studies were performed in compliance with the animal policies of the Max Planck Institute of Experimental Medicine, and were approved by the German Federal State of Lower Saxony (LAVES).

Note that full information on the approval of the study protocol must also be provided in the manuscript.
